# Intermediate filament protein vimentin propels intrahepatic lipid accumulation in insulin-resistant mice

**DOI:** 10.1016/j.jbc.2026.113265

**Published:** 2026-06-19

**Authors:** Priya Rawat, Shilpa Thakur, Kajal Jaswal, Aniket Sen, Agnimitra Biswal, Budheswar Dehury, Prosenjit Mondal

**Affiliations:** 1 School of Biosciences and Bioengineering, Indian Institute of Technology Mandi, Mandi, Himachal Pradesh, India; 2 Department of Biological Sciences, Indian Institute of Science Education and Research Berhampur, Berhampur, India; 3 Department of Bioinformatics, Manipal School of Life Sciences, Manipal Academy of Higher Education, Manipal, Karnataka, India

**Keywords:** Kupffer cells, hepatocytes, *de novo* lipogenesis, insulin resistance, vimentin, AMPK

## Abstract

Insulin resistance is known for promoting lipid accumulation in hepatocytes mainly by enhancing hepatic *de novo* lipogenesis (DNL), thereby playing a role in the development and progression of metabolic dysfunction-associated fatty liver disease (MAFLD). Nonetheless, the paradox persists that hepatocytes sustain enhanced DNL activity despite compromised insulin signaling. The molecular mechanisms that sustain this lipogenic drive in the presence of insulin resistance remain inadequately elucidated, highlighting a significant deficiency in our comprehension of MAFLD etiology. In this study, we observed that a high-fat diet (HFD) increases the expression and release of Vimentin from Kupffer cells (KCs), which, in turn, cross-talks with the hepatocytes' IGF1 receptor to sequester LKB1 in the nucleus and repress AMPK activation. Serum vimentin levels were elevated in patients with MAFLD and the HFD-induced MAFLD mouse model. Interestingly, both KC depletion and the Vimentin knockdown attenuated intrahepatic fat accumulation. Moreover, supplementation of recombinant Vimentin in KC-depleted HFD mice resulted in increased intrahepatic lipid accumulation, suggesting a plausible role of Vimentin in the pathophysiology of MAFLD. Mechanistically, HFD increased Vimentin expression. Upon secretion from KCs, Vimentin interacts with IGF1 receptor of the hepatocytes, leading to reduced hepatic AMPK activation and enhanced DNL. Thus, the study shows that obesity promotes hepatic DNL in the insulin-resistant state *via* Vimentin, a hitherto unknown organokine.

Metabolic dysfunction-associated fatty liver disease (MAFLD) is closely associated with obesity and insulin resistance. There are theories concerning the accumulation of fat and the onset of insulin resistance. The first theory suggests that the excess build-up and accumulation of free fatty acids and triglycerides (TG) in the liver leads to hepatic insulin resistance, further activating the immune system and inflammation (1, 2, 3, 4). On the other hand, the second theory proposes that the initial activation of the immune system primarily involves liver macrophages, which initiate acute inflammation and subsequently lead to hepatic lipid accumulation and insulin resistance (5, 6, 7). Recent evidence suggests that intrahepatic crosstalk between hepatocytes and other hepatic cells can regulate hepatocytes' metabolism, supporting the second theory (8, 9).

The liver comprises two different cell populations: liver parenchymal cells, which constitute 60% of hepatocytes, and liver non-parenchymal cells, which comprise 30 to 35%. Among those liver non-parenchymal cells, residential Kupffer cells (KCs) constitute 35% (10). These specialized tissue macrophages protect the liver against incoming threats and play a pivotal role in inflammation and hepatic homeostasis. KCs' activation and inflammation induction have been reported as a well-known event in liver-associated metabolic diseases (11, 12). A recent report shows that KCs can sense free fatty acid availability, induce inflammation, and regulate hepatic lipid metabolism in a high-fat diet (HFD) (13).

Hepatic lipid accumulation is a multifaceted process influenced by diverse stimuli, ranging from nutritional cues to pathological conditions. In the context of insulin resistance, under conditions of heightened nutrient availability, hepatocytes respond by intensifying *de novo* lipogenesis (DNL), thereby contributing to lipid accumulation in the liver. Moreover, the activation of inflammation in response to increased nutrient overload underscores the intricate relationship between KCs and hepatocytes. However, the precise role of the KCs in regulating hepatic lipid metabolism remains to be elucidated. Under normal physiological conditions, insulin promotes DNL in hepatocytes. However, during insulin resistance (when insulin signaling is blunted) and accompanied by hyperinsulinemia (HI), the promotion of hepatic DNL continues, which is not fully elucidated (14). In this study, we discovered that Vimentin secreted by KCs promotes DNL in the presence of insulin resistance in the liver.

We previously reported that serine/threonine kinase 38 (STK38) serves as a node linking heightened nutrient availability to hepatic inflammation, ultimately leading to hepatic lipid accumulation and insulin resistance (15). Here, we explored the role of KCs’ STK38 in intrahepatic crosstalk between KCs and hepatocytes, which can modulate lipid metabolism and lead to the onset and progression of MAFLD.

The present study found that KC-derived Vimentin promotes intrahepatic lipid accumulation in an insulin-resistant state by altering hepatocyte lipid metabolism. We depleted KC’s STK38 to explore the release and function of Vimentin in KC-HCs' communication in the dietary model of MAFLD. Vimentin is an intermediate filament protein that contributes to various cellular processes, including migration, morphology, plasticity, and organelle anchoring. Vimentin is expressed in various mesenchymal cell types, including macrophages, fibroblasts, endothelial cells, melanocytes, Schwann cells, and lymphocytes. Although Vimentin is a cytoskeletal protein, multiple studies have shown that it can be expressed on cell surfaces or secreted by macrophages and microvascular endothelial cells, suggesting an unconventional role in cellular dynamics (16). However, the regulation of its secretion and its role systemic Vimentin in developing and progressing MAFLD and associated pathologies remains largely unknown. We show that KC’s STK38 regulates Vimentin release, suggesting its antagonism improves intrahepatic fat and glycemia in MAFLD. Our data also indicate that Vimentin levels are increased in the serum of MAFLD patients and in the HFD-induced MAFLD mouse model. These results suggest that KC’s released Vimentin may serve as a therapeutic target for treating insulin resistance-mediated fatty liver pathophysiology.

## Results

### KCs STK38 in regulating hepatic lipid accumulation

**Figure 1:**
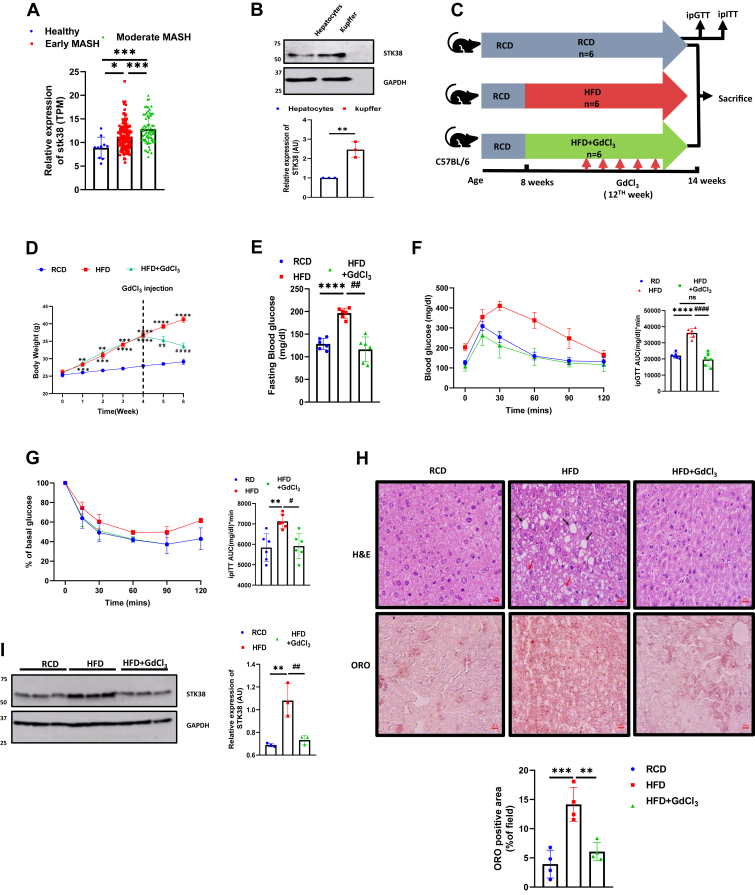
**Kupffer cells’ STK38 promotes hepatic steatosis.***A*, transcript profile of *STK38* in the liver of healthy and MASH subjects. (healthy (n) = 10, early MASH (n) = 137), moderate MASH (n) = 69). *B*, qualitative and quantitative analysis of STK38 protein level normalized to GAPDH in primary hepatocytes and Kupffer cells (n = 3). *C*, schematic description of dietary regimen and treatment of C57BL/6 male mice (n = 6 in each group). *D*, graphical representation of body weight (n = 6 in each group). *E*, fasting blood glucose level of RCD, HFD, and HFD + GdCl_3_ mice was measured on 14th week after 6 h of fasting (n = 6 in each group). *F* and *G*, mice were fasted overnight. A total of 2 g/kg glucose and 0.5 U/kg insulin were injected intraperitoneally, and GTT and ITT were performed at 0,15, 30, 60, 90, and 120 min and represented as AUC, respectively (n = 6 in each group). H, H&E staining and ORO staining of RCD, HFD, and HFD + GdCl3 depicting macrovesicular steatosis (*black arrow*): large lipid droplets are present in hepatocytes and microvesicular steatosis (*red arrow*): small lipid droplets are present in hepatocytes at 40 X. Oil Red O positive lipid area was quantified using ImageJ by applying a uniform threshold across all images and calculating the percentage of positive area relative to the total field. *I*, qualitative and quantitative analysis of STK38 protein level normalized to GAPDH in the liver of RCD, HFD, HFD + GdCl_3_ mice (n = 3 each). Data are represented as mean ± SD, and n represents biological replicates. Comparisons between human subjects were performed using one-way ANOVA followed by Bonferroni’s multiple comparisons test. Statistical significance is indicated as follows: ∗*p* < 0.05; ∗∗∗/#*p* < 0.001. Comparisons between samples in panel (*B*) were conducted using an unpaired Student’s *t* test (∗*p* < 0.05). Panel (D) was analyzed using two-way ANOVA followed by Bonferroni’s multiple comparisons test. Comparisons across treatment groups in panels (*E*, *F*, *G*, and *H*) were analyzed using one-way ANOVA followed by Bonferroni’s multiple comparisons test to determine differences between specific groups. For panels (*D*, *E*, *F*, *G*, and *H*), ∗ indicates statistically significant differences between RCD *versus* HFD, and # indicates statistically significant differences between HFD *versus* HFD + GdCl_3_. Significance levels are denoted as: ns: not significant (*p* > 0.05), ∗^/#^*p* < 0.05, ∗∗^/#^*p* < 0.01,∗∗∗^/#^*p* < 0.001, and ∗^/#^*p* < 0.0001. GdCl3, gadolinium(III) chloride; HFD, high-fat diet; MASH, metabolic dysfunction-associated steatohepatitis; RCD, regular chow diet; STK38, serine/threonine kinase 38.

Our previous study in a murine model demonstrated the role of STK38 in promoting hepatic insulin resistance and lipodystrophy through activation of the TBK1/NF-κB signaling pathway, thereby contributing to the onset of MAFLD. To evaluate its relevance in human disease, we analyzed the expression of STK38 genes using publicly available hepatic transcriptome data from healthy and metabolic dysfunction-associated steatohepatitis (MASH) patients. The transcript level of *STK38* was significantly higher in early MASH and moderate MASH than in healthy subjects (Fig. 1*A*). Consistent with this, our murine study showed that the HFD-fed mice had an increased hepatic F4/80^+^ population along with more fat accumulation and insulin resistance in the liver; however, depletion of hepatic STK38 in HFD mice exhibited a decreased hepatic F4/80^+^ population (Fig. S1*A*), reduced liver fat and improved insulin sensitivity (15). Therefore, to better understand the role of STK38 in intrahepatic communication, we investigated its distribution within the liver, revealing a higher abundance in KCs than HCs (Fig. 1*B*). Based on these cues, we examined the potential role of KCs in regulating anabolic processes in hepatocyte lipid metabolism under an insulin-resistant state. Activated KCs or residential hepatic macrophages (RHMs) play a crucial role in the development of MAFLD (17, 18). To examine the interaction between KCs and hepatocytes in MAFLD, we depleted KCs in the livers of HFD mice using Gadolinium(III) chloride GdCl_3_ treatment (Fig. 1*C*) (5, 19). We observed a significant reduction in body weight in GdCl3-treated mice compared with HFD mice (Fig. 1*D*). We found elevated fasting blood glucose (FBG) levels in HFD mice compared to the regular chow diet (RCD) group, and surprisingly, GdCl_3_-treated mice showed lower FBG levels than the HFD mice (Fig. 1*E*). In addition, to gain insight into systemic glucose and insulin sensitivity, we performed intraperitoneal glucose tolerance tests (ipGTT) and intraperitoneal insulin tolerance tests (ipITT) (Fig. 1, *F* and *G*). The GdCl_3_ mice were more glucose-tolerant and insulin-sensitive than HFD mice. Our data indicate that KCs and RHMs play a significant role in regulating hepatic glucose metabolism. To confirm that the effect of GdCl_3_ treatment is truly dependent on RHM reduction, we performed immunohistochemistry to verify a reduced RHM population. Our results confirmed a reduction in F4/80+ cells in GdCl3-treated mice compared to HFD mice (Fig. S1*B*). We next asked whether KC depletion influences lipid metabolism in hepatocytes of HFD-fed mice and assessed its impact on intrahepatic lipid accumulation. We performed H&E and ORO staining of all three groups; we observed increased hepatic lipid accumulation characterized by microvesicular and macrovesicular steatosis in HFD mice compared to the RCD group, which was alleviated in HFD mice treated with GdCl_3_ (Fig. 1*H*). These findings suggest that depleting KCs is sufficient to reduce intrahepatic lipid accumulation. Furthermore, GdCl3-treated HFD mice exhibited remarkably reduced hepatic STK38 expression, substantiating the abundance of STK38 in KCs (Fig. 1*I*). This suggests a possible role of KCs' STK38 in hepatic insulin resistance and lipid metabolism.

### STK38 dictates Kupffer-hepatocyte crosstalk and regulates hepatic lipid accumulation in *via* LKB1-AMPK axis

**Figure 2:**
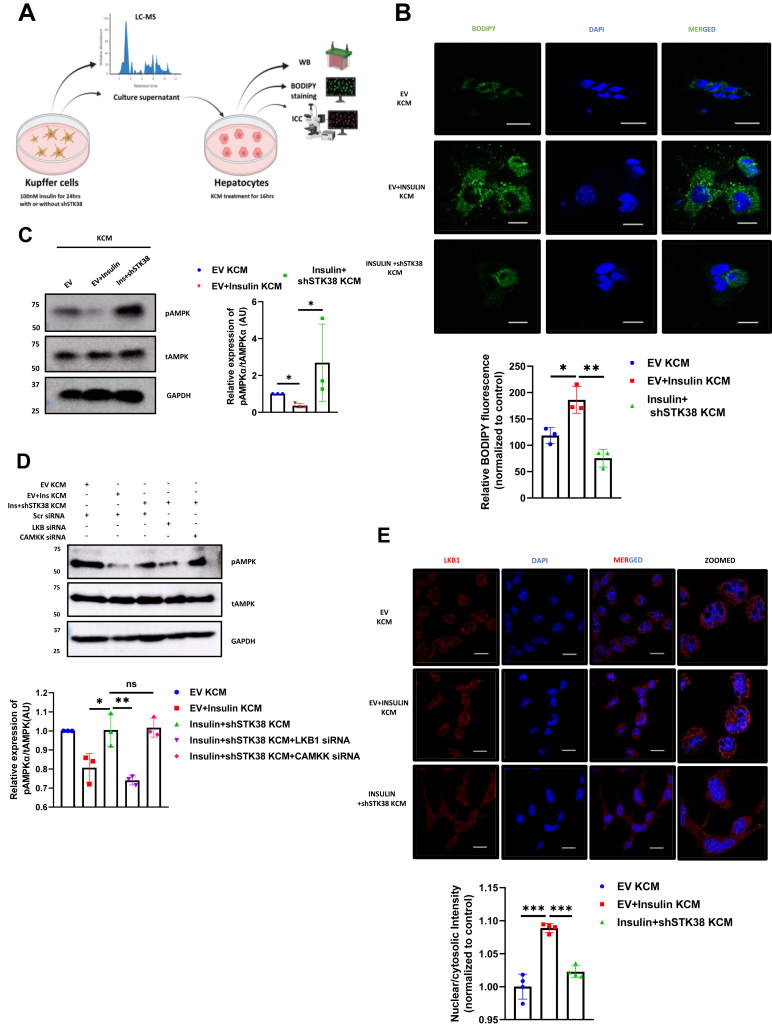
**Kupffer cell-derived STK38 regulates hepatocyte lipid accumulation *via* LKB1-AMPK signaling.***A*, schematic representation of the overall experimental design, including collecting conditioned media from Kupffer cells and treatment to hepatocytes (primary hepatocytes and AML-12) and secretome analysis (n = 3). *B*, BODIPY staining in primary hepatocytes after treatment of Kupffer cell-conditioned media treated with 100 nM insulin (HI) with or without STK38 for 16 h. BODIPY fluorescence intensity was measured as integrated density using ImageJ, normalized to cell number, and expressed relative to the control. The scale bar represents 20 μm. *C*, the phosphorylation of AMPKα normalized to total-AMPKα in primary hepatocytes cells and expressed relative to the control group. Expression was measured after treatment with control + EVC (empty vector control), HI + EVC, and HI + shSTK38 KCM for 16 h and analyzed by Western blot and quantified using ImageJ (n = 3). *D*, qualitative and quantitative expression of phospho-AMPKα at T172 normalized to total-AMPKα after LKB1 and CAMKK knockdown and treatment of control + EVC, insulin + EVC, and insulin + shSTK38 KCM for 16 h in HepG2 cells (n = 3). Scrabled siRNA (SCsiRNA) was used as a transfection control in control + EVC, insulin + EVC, and insulin + shSTK38 KCM treated cells. The ratio of pAMPK to total AMPK is expressed relative to the control group. *E*, nuclear and cytosolic translocation of LKB1 after treatment of insulin-treated KCM and insulin + shSTK38 KCM for 16 h of treatment was observed after ICC in AML12Cells. Nuclear and cytosolic regions were defined based on DAPI staining, and LKB1 fluorescence intensity was quantified using ImageJ. The nuclear-to-cytosolic (N/C) ratio of LKB1 was calculated for each cell and normalized to the control. The scale bar represents 20 μM. Data are represented as Mean ± SD. *n* represents biological replicates. Comparisons across the treatments (*B*, *C*, *D*, and *E*) were performed using one-way ANOVA followed by Bonferroni’s multiple comparisons test. Statistical significance is indicated as: ns: not significant (*p* > 0.05), ∗*p* < 0.05, ∗∗*p* < 0.01, and ∗∗∗*p* < 0.001. CaMKK, Ca2+/calmodulin-dependent protein kinase kinase; DAPI, 4',6-diamidino-2-phenylindole; EVC, empty vector control; HI, hyperinsulinemia; KCM, Kupffer cells' conditioned media; STK38, serine/threonine kinase 38.

Our findings suggest that KCs might crosstalk with HCs, leading to a hepatic metabolic imbalance in the HFD mice. To establish the mechanistic link between aberrant hepatic lipid metabolism and lipogenesis with KCs secretory factors in insulin resistance conditions, we designed an *in vitro* system with mouse primary KCs and HCs. We isolated mouse primary KCs followed by treatment with 100 nM insulin (HI) (Fig. 2*A*). The use of HI conditions is based on other studies indicating that elevated insulin levels could potentially play a role in fostering insulin resistance and the onset of MAFLD (20). We observed an upregulation of STK38 expression in HI-treated KCs (Fig. S1*C*). Upon administration of HI-treated KCs’ conditioned media (KCM) to the HCs, a noticeably enhanced intrahepatic lipid accumulation was evident compared to the control. Later, to explore the role of KC-specific STK38 in hepatic lipid accumulation, we deplete STK38 in KCs using shRNA (Fig. S1*C*). Interestingly, HCs exposed to KCM from STK38-depleted, HI-treated KCs showed fewer intracellular lipid droplets compared to those exposed to KCM from HI-treated KCs (Fig. 2*B*). Next, to determine the possible molecular mechanism by which STK38-depleted KCM attenuates intrahepatic lipid accumulation, we measured the impact of KCM on AMPK activation in HCs. AMPK activation lowers hepatic TG content by inhibiting ACC, thereby reducing DNL. Phosphorylation at the Thr172 residue of AMPKα is used as a surrogate marker for its activation. Our data suggested that HI-treated KCM significantly diminished AMPKα phosphorylation of primary hepatocytes compared to the control. In contrast, KCM collected from HI-treated KCs after STK38 depletion restored the phosphorylation of AMPKα (Fig. 2*C*).

Next, to verify the mechanism of AMPK activation by STK38-depleted, HI-treated KCM, we investigated the role of two major upstream regulators of AMPK, LKB1 and Ca2+/calmodulin-dependent protein kinase kinase beta (CaMKKβ), which are responsible for phosphorylating AMPKα at Thr172 (21, 22). For this, HepG2 cells were transfected with either LKB1- or CaMKKβ-specific siRNA and then treated with HI-KCM, with or without STK38 depletion, to assess AMPKα phosphorylation at Thr172. As expected, HI-KCM reduced AMPKα phosphorylation compared to Con-KCM. In contrast, STK38-depleted HI-KCM markedly increased p-AMPK levels in HepG2 cells. Notably, this induction was abolished in LKB1 siRNA-transfected cells but not in CaMKKβ siRNA-transfected cells, indicating that LKB1 but not CaMKKβ is required for this effect (Fig. 2*D*). Next, we examined LKB1 localization in AML12 cells after KCM treatment. We observed that HI-KCM caused LKB1 to remain sequestered in the nucleus, whereas STK38-depleted HI-KCM promoted its translocation from the nucleus to the cytosol (Fig. 2*E*).

Together, our findings suggest that KCs contribute to hepatocyte lipid accumulation in hyperinsulinemic conditions *via* STK38-mediated signaling. Depleting STK38 in KCs mitigates the pro-steatotic effect. HI- treated KCM reduced hepatocyte AMPK activity *via* nuclear sequestration of LKB1. The depletion of STK38 promotes the cytosolic translocation of LKB1, which in turn enhances AMPK activation and inhibits lipid accumulation. These findings highlight the significant role of KC-derived STK38 in perturbing hepatic lipid homeostasis.

### Vimentin secretion from KCs drives hepatic lipid accumulation *via* IGF1 receptor (IGF-1R)-mediated AMPK suppression

**Figure 3:**
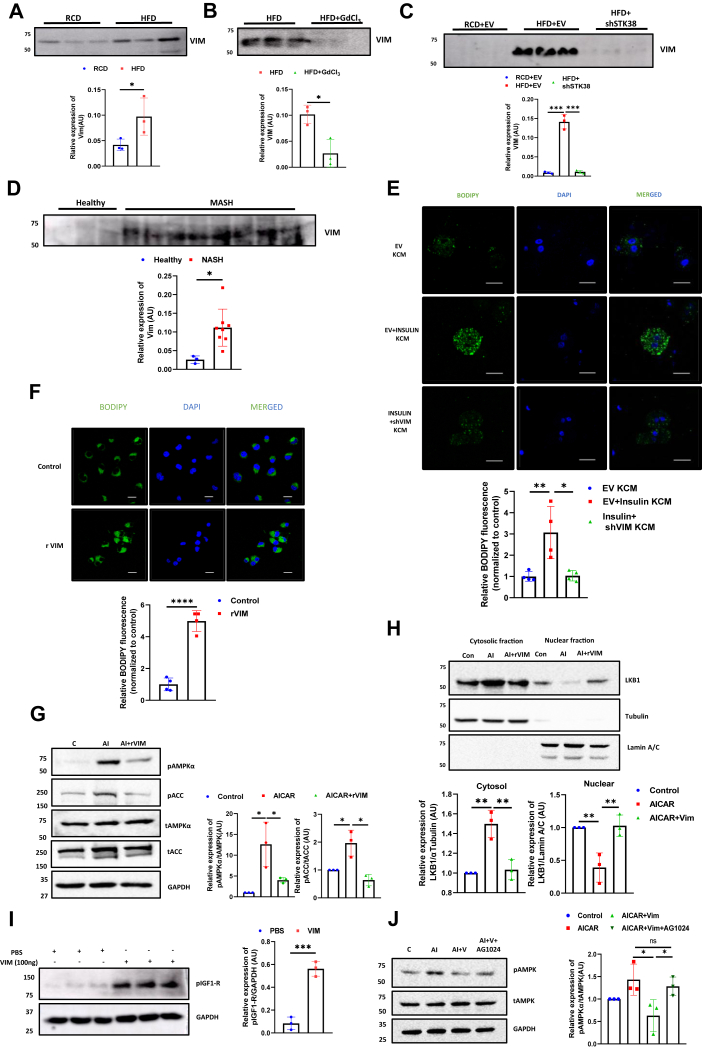
**KC-derived vimentin promotes hepatic lipid accumulation *via* IGF1R/AMPK pathway.***A*, qualitative representation of the vimentin protein level normalized to total protein in the serum of RCD and HFD mice (n = 3 each). *B*, qualitative representation of the vimentin protein level normalized to total protein in the serum of HFD mice and HFD + GdCl_3_ mice (n = 3 each). *C*, qualitative representation of the vimentin protein level normalized to total protein in the serum of RCD + EVC (empty vector control), HFD + EVC, and HFD + shSTK38mice (n = 3 each). *D*, qualitative and quantitative expression of vimentin normalized to total protein in the serum of healthy (n = 3) and MASH (n = 8) human subjects. *E*, BODIPY staining in primary hepatocytes after control, insulin, and insulin + shVimentin Kupffer-conditioned media treatment after 16 h. BODIPY fluorescence intensity was measured as integrated density using ImageJ, normalized to cell number, and expressed relative to the control. The scale bar represents 20 μm. *F*, BODIPY staining in AML-12 cells after treatment with 100 ng rVim for 16 h. BODIPY fluorescence intensity was measured as integrated density using ImageJ, normalized to cell number, and expressed relative to the control. The scale bar represents 20 μm. *G*, qualitative and quantitative expression of phosphorylated AMPKα (T172) and ACC (S79) normalized by total-AMPKα and ACC in HepG2 cells after being treated with AICAR and AICAR + rVim for 16 h (n = 3). The ratio of pAMPK to total AMPK and pACC to total-ACC is expressed relative to the control group. *H*, qualitative and quantitative representation of the level of LKB1 in the nuclear/cytosolic extract of control, AICAR, and AICAR + rVim-treated AML12 cell (n = 3). Nuclear and cytosolic LKB1 levels were normalized to lamin A/C and α-tubulin, respectively, and expressed relative to the corresponding control group. *I*, phosphorylation of IGF1-R at Tyr1135/1136 normalized to GAPDH in HepG2 cells after VIM treatment for 15 min (n = 3). *J*, phosphorylation of AMPKα normalized by total-AMPKα in HepG2 cells after being treated with AICAR, AICAR + rVim, and AICAR + rVim + AG1024 for 16 h, where 700 nM AG1024 was pretreated for 32 h before the treatment of AICAR and rVim, and analyzed by Western blot and quantified using ImageJ. The ratio of pAMPK to total AMPK is expressed relative to the control group. Data are represented as mean ± SD. *n* represents biological replicates. Comparisons between samples in panel (*A*, *B*, *C*, *D*, *F* and *I*) were conducted using an unpaired Student’s *t* test. Significance levels are denoted as: ns: not significant (*p* > 0.05), ∗*p* < 0.05,∗∗*p* < 0.01, and ∗∗∗*p* < 0.001. Comparisons across the treatments (*E*, *G*, *H* and *J*) were performed using one-way ANOVA followed by Bonferroni’s multiple comparisons test. Statistical significance is indicated as: ns: not significant (*p* > 0.05), ∗*p* < 0.05, ∗∗*p* < 0.01, and ∗∗∗*p* < 0.001. EVC, empty vector control; GdCl_3_, gadolinium(III) chloride; HFD, high-fat diet; KC, Kupffer cell; IGF1R, IGF1 receptor; RCD, regular chow diet; rVim, recombinant Vimentin.

Next, we investigated the factors secreted by HI-treated KCs that could induce hepatic lipid accumulation. Proteomic analysis of the KCM identified Vimentin as a differentially expressed candidate protein (Table S1). To explore the level of Vimentin in pathophysiological conditions, we investigated the level of Vimentin in 6-weeks HFD-fed mice. We observed higher Vimentin levels in the serum of HFD mice compared to RCD mice (Figs. 3*A* and S2*A*). Moreover, depletion of KCs using GdCl_3_ led to decreased hepatic and serum levels of Vimentin, indicating that KCs may be the predominant source of systemic Vimentin (Figs. 3*B* and S2*B*). Next, we validated STK38's role in Vimentin release under HFD conditions. We knocked down STK38 in HFD mice and measured serum Vimentin levels. We observed enhanced Vimentin in the serum of HFD mice compared to RCD mice, while STK38 knockdown leads to reduced Vimentin level in the mouse serum compared to HFD mice (Figs. 3*C* and S2, *C* and *D*). This observation indicates that STK38 upregulates the expression and secretion of Vimentin in HFD-induced MAFLD conditions. Next, we collected the blood samples from a cohort of biopsy-proven MASH patients and age and sex-matched control subjects (Table S2). Consistent with our data, circulatory Vimentin levels were considerably higher in MASH patients than in healthy subjects (Fig. 3*D* and S2*E*).

To establish the causal links between aberrant Vimentin release from KCs and impaired lipid metabolism in HCs, we knocked down Vimentin in HI-treated KCs and supplemented primary hepatocytes with the respective KCM. Interestingly, we observed reduced lipid accumulation in HCs supplemented with Vimentin-depleted HI-treated KCM compared to HCs supplemented with HI-treated KCM (Fig. 3*E*).

To validate vimentin’s adverse impact on hepatic lipid metabolism, AML12 cells were treated with recombinant Vimentin (rVim), which led to increased hepatic lipid accumulation. (Fig. 3*F*). Next, we aim to investigate the effect of rVim on AMPK activation. We visualized the phosphorylation of the Thr172 residue of AMPK (p-AMPK), a surrogate marker of AMPK activity. rVim treatment significantly attenuated AICAR-mediated AMPK activation. In parallel, phosphorylation of ACC at Ser79 was also reduced, which was consistent with increased lipid accumulation in hepatocytes following rVim treatment (Fig. 3*G*). To further assess the role of Vimentin in AICAR-dependent LKB1 activation, AML12 cells were treated with AICAR and rVim, followed by nuclear/cytosolic fractionation. As expected, AICAR treatment enhanced the cytosolic localization of LKB1. In contrast, rVim treatment caused LKB1 to remain sequestered in the nucleus, even in the presence of AICAR (Fig. 3*H*). These findings showed that Vimentin diminished AMPK activity by nuclear sequestration of LKB1 and promoted DNL, thereby inducing intrahepatic lipid accumulation. Consequently, we sought to determine the mechanism by which Vimentin impairs hepatic lipid metabolism. Literature suggests that Vimentin can interact with IGF1 receptor (IGF-1R), which is abundantly expressed in the liver and regulates nutrient metabolism pathways (23, 24). To specifically investigate the functional role of IGF-1R on hepatocytes in mediating Vimentin action, we examined the phosphorylation of IGF-1R at Tyr1135/1136, which is crucial for its activation. After treatment with 100 ng of rVim for 15 min, we observed increased phosphorylation of IGF-1R at Tyr1135/1136 compared to the PBS control (Fig. 3*I*), confirming Vimentin-dependent IGF-1R activation. IGF1 is a well-known IGF-1R ligand that binds to the receptor and regulates various cellular signaling pathways. Although the role of IGF1/IGF-1R/AMPK signaling is not well understood in the liver, a study reveals that IGF1 binding to IGF-1R activates AMPKα in neural cells (25). We conduct *in silico* research to better understand how IGF1 and Vimentin bind to IFG-1R, activating various signaling pathways. We performed a docking study using HADDOCK and discovered that Vimentin binds to a distinct region of IGF-1R compared to the traditional ligand, IGF1 (Fig. S2, *F* and *G*; Tables S3 and S4). To specifically investigate the functional role of IGF-1R in mediating Vimentin's effects on hepatocyte nutrient metabolism, we pretreated HepG2 cells with the IGF-1R antagonist AG1024, followed by rVim treatment in the presence of AICAR (26). Data suggested that rVim treatment significantly reduced AICAR-mediated AMPK activation, whereas, pharmacological inhibition of IGF-1R abolished the rVim-induced reduction of AICAR-mediated AMPKα phosphorylation, indicating that Vimentin may suppresses AICAR-mediated AMPK activity in hepatocytes through IGF-1R engagement. (Fig. 3*J*). AG1024 mediated IGF-1R antagonization was confirmed by a marked reduction in IGF-1R phosphorylation (Fig. S2*H*). Together, our findings demonstrate that systemic Vimentin can directly disrupt hepatocyte lipid metabolism by inhibiting AMPK activation through the nuclear sequestration of LKB1 and by activating IGF-1R.

### Vimentin exacerbates hepatic lipid accumulation in KC-depleted HFD mice

**Figure 4:**
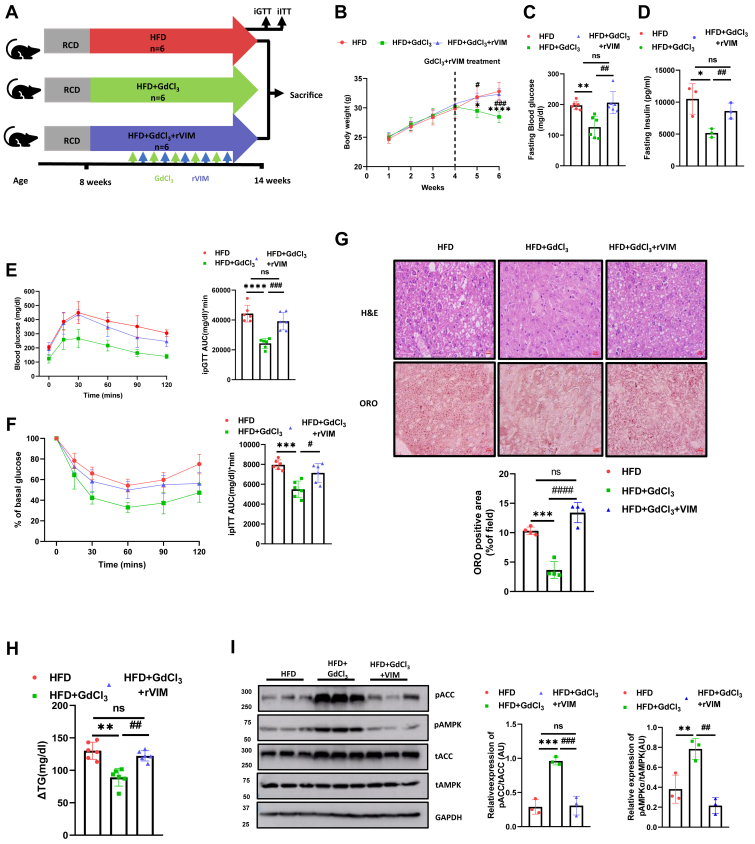
**Vimentin exacerbates lipid accumulation in Kupffer cell-depleted HFD mice.***A*, schematic description of dietary regimen and treatment of C57BL/6 male mice (n = 6 in each group). *B*, graphical representation of bodyweight (n = 6 in each group). *C* and *D*, fasting blood glucose and insulin levels of HFD, HFD + GdCl3, and HFD + GdCl_3_+rVim mice were observed after 6 h of fasting on the 14th week (n = 6). *E* and *F*, mice were fasted overnight. A total of 2 g/kg glucose and 0.5 U/kg insulin were injected intraperitoneally, and GTT and ITT were performed at 0, 15, 30, 60, 90, and 120 min and represented as AUC, respectively (n = 6 in each group). *G*, H&E staining and ORO staining in the liver of HFD, HFD + GdCl3, and HFD + GdCl_3_+rVim mice at 40 X. Oil Red O positive lipid area was quantified using ImageJ by applying a uniform threshold across all images and calculating the percentage of positive area relative to the total field. *H*, hepatic TG level in HFD, HFD + GdCl_3_, and HFD + GdCl_3_+rVim treated mice (n = 6 in each group). *I*, phosphorylation of AMPKα (T172) and ACC (S79) normalized to their respective total protein levels in the livers of HFD, HFD + GdCl_3_, and HFD + GdCl_3_+rVim mice, analyzed by Western blot and quantified using ImageJ. (n = 3 in each group). Data are represented as mean ± SD. *n* represents biological replicates. Panel (*B*) was analyzed using two-way ANOVA followed by Bonferroni’s multiple comparisons test. Comparisons between samples in panel (*C D*, *E*, *F*, *G*, *H*, and *I*) were conducted using one-way ANOVA followed by Bonferroni’s multiple comparisons test. ∗ indicates statistically significant differences between HFD *versus* HFD + GdCl_3_, and # indicates statistically significant differences between HFD + GdCl_3_*versus* HFD + GdCl_3_+rVIM. Statistical significance is indicated as: ns: not significant (*p* > 0.05), ∗^/#^*p* < 0.05, ∗∗^/#^*p* < 0.01,∗∗∗^/#^*p* < 0.001, and ∗∗∗∗^/####^*p* < 0.0001. GdCl_3_, gadolinium(III) chloride; HFD, high-fat diet; rVim, recombinant Vimentin; TG, triglycerides.

The finding that Vimentin mediated intrahepatic crosstalk in the preclinical MAFLD mouse model prompted us to explore the effects of chronic Vimentin exposure. Therefore, after 6 weeks of HFD diet feeding, treated with GdCl_3._, a subset of mice was intraperitoneally injected with saline or rVim (1 μg/mouse) every other day for 2 weeks (Fig. 4*A*). As expected, HFD-fed mice gained substantial body weight, whereas depletion of KCs-protected mice from HFD-induced body weight gain; however, rVim-treated mice displayed similar weight gain as HFD mice (Fig. 4*B*). In addition, we found subdued fasting glucose and insulin levels in the GdCl_3_-treated group, whereas rVim supplementation enhanced FBG and insulin levels in the GdCl_3_-treated mice (Fig. 4, *C* and *D*). Furthermore, we found that the GdCl_3_ treatment failed to improve glucose and insulin intolerance in the rVim-treated HFD group compared to only GdCl_3_-treated HFD mice (Fig. 4, *E* and *F*). The ICC confirmed the depletion of KCs from the liver section of the GdCl3-and GdCl3+rVim-treated HFD groups (Fig. S3*A*). Furthermore, we couldn’t find any change in STK38 expression among the rVim-treated GdCl_3_ mice and the GdCl_3_ mice, suggesting that STK38 plays a role upstream of vimentin (Fig. S3*B*). Moreover, we performed H&E and ORO staining to assess histopathological changes and lipid content across all three groups (Fig. 4*G*). Besides, the GdCl_3_-treated HFD group showed reduced hepatic TG content compared to the HFD group, while the rVim-treated HFD-GdCl_3_ group displayed higher hepatic TG content than the GdCl_3_ group (Fig. 4*H*). We noticed hepatic lipid accumulation was higher in the rVim-treated HFD-GdCl_3_ mice than in the HFD-GdCl_3_ group. In a similar direction, we investigated the phosphorylation of ACC at S79 and AMPK at T172, which were reduced in rVim-treated HFD + GdCl_3_ mice compared to the GdCl_3_-treated HFD group. We visualized the phosphorylation of the Thr172 residue of AMPK (p-AMPK), a surrogate marker of AMPK activity (Fig. 4*I*). This observation suggests that in HFD conditions, intrahepatic crosstalk between KCs and HCs leads to impaired DNL in HCs. In the HFD mice liver, the increased release of Vimentin from activated KCs causes enhanced lipid accumulation in hepatocytes. Findings demonstrate that chronic exposure to Vimentin could accelerate the pathogenesis of MAFLD by enhancing DNL. Our data strongly suggest that HFD-induced fatty liver impairs hepatocytes' lipid metabolism *via* KC’s Vimentin. Diminution of circulatory Vimentin would improve metabolic outcomes by enhancing hepatocyte AMPK activity and glucose tolerance in insulin resistance-induced DNL and, in turn, MAFLD.

## Discussion

We identified Vimentin as a novel messenger mediating hepatocyte lipid metabolism using complementary *in vitro, ex vivo,* and *in vivo* experiments. We show that treatment with HI *in vitro* or by feeding HFD in mice led to upregulation of KC’s Vimentin levels in an STK38-dependent way. Augmented systemic Vimentin can perturb LKB1 subcellular localization and inhibit AMPK activation in hepatocytes, leading to enhanced DNL. KC-deficient (GdCl_3_-treated) HFD mice resulted in reduced serum Vimentin levels, improved glucose tolerance, insulin sensitivity, and intrahepatic lipid accumulation *in vivo*, uncovering the KCs as a site of regulated Vimentin production and release. This data provide the first evidence that secreted Vimentin is a critical contributor to intrahepatic lipid accumulation in insulin-resistant individuals *via* KCs-HCs crosstalk.

Vimentin is an intermediate filament protein with a single subunit that performs structural functions for the cell (27). Beyond maintaining cell integrity, it plays a role in cellular communication by secreting from fibroblasts, endothelial cells, and activated macrophages. Specifically, it is reported to be abundantly expressed in human monocytes and activated macrophages (28). Our *in vitro* study found a fascinating link between the release of Vimentin from the KCs and the regulation of the metabolic function of hepatocytes. Consistently enhanced Vimentin expression in the serum of MASH patients and the HFD mice compared to RCD mice confirms the potential involvement of Vimentin in inducing metabolic disease.

In a murine model, we found that STK38 is more abundant in the KCs than in the hepatocytes. The elevation of STK38 in diet-induced obese mice is correlated with the release of Vimentin, a protein associated with inflammation. Moreover, the conditioned media from the hyperinsulin-treated KCs caused significant lipid accumulation in the hepatocytes. Interestingly, this effect was remarkably reduced with the condition media where STK38 and Vimentin were silenced in KCs, suggesting a critical role of KC’s STK38 in regulating the lipid metabolism of hepatocytes *via* releasing Vimentin. We also investigated the transcriptomic profile of *STK38* in the healthy and MASH subjects, where we found that the progression of MASH is positively correlated with liver STK38 expression.

Several reports suggest that Vimentin is a soluble protein that works as a ligand. However, its receptors remain poorly understood. One report indicates that Vimentin can act as a ligand to IGF-1R, consequently facilitating axonal growth (23). Our study also reinforces the previous observation, where using pharmacological blockade of IGF-1R prevents Vimentin from inhibiting AMPKα phosphorylation and substantiates that Vimentin may act on hepatocytes *via* IGF-1R. Our results support the role of Vimentin as an organokine of MAFLD development and progression, at least in part, through IGF-1R-dependent ways. In silico analysis reveals that Vimentin has a distinct binding location on IGF-1R compared to IGF1, which may activate a separate signaling pathway, hence modulating hepatic DNL.

In addition to exploring molecular and cellular events in KC-driven Vimentin expression and its adverse impact on hepatocyte lipid metabolism, the present study could also be relevant for future translational medicine. First, elevated Vimentin expression levels are associated with several diseases, *viz.* cancer, Alzheimer’s disease, and inflammatory diseases (29). Now, we have unraveled its pathogenetic role in obesity-induced MAFLD. Although the exact mechanism of how Vimentin is secreted from the KC is not well understood, elevated serum levels of Vimentin may serve as a biomarker in identifying patients with a heightened risk of developing MAFLD in obese individuals.

In conclusion, our findings provide a mechanism to explain how obesity promotes hepatic DNL in the insulin-resistant state. This showed that insulin resistance induced by a HFD triggers two significant responses: first, it induces KC’s STK38 expression, leading to enhanced Vimentin release. Vimentin binds to hepatocytes' IGF-1R, prompting the nuclear retention of LKB1, boosting hepatic lipogenesis, and the accumulation of intrahepatic lipids by inactivating AMPK activity. This discovery holds significant therapeutic promise in mitigating obesity-induced hepatic insulin resistance, associated fatty liver disease, and dyslipidemia.

## Experimental procedures

### Mice

All experiments were approved by the Institutional Animal Ethics Committee at the Indian Institute of Technology Mandi, the *Committee for Control and Supervision of Experiments on Animals (CCSEA),* the Ministry of Environment, Forest and Climate Change, and the Government of India. C57BL/6 mice (Male, 8-week-old) were procured from the institutional animal facility of IISER Mohali, India, and acclimatized for 7 days at 23 °C and 50 to 60% humidity with a 12-h light and 12-h dark cycle. and free access to food and water. For the first study, mice were subjected to a RCD and a HFD. The RCD group was fed RCD containing ∼14% calories from fat [Protein 20%, Fat 14.725%, carbohydrate 16.149%] (5L79 Lab diet). The HFD group was fed a diet containing 60% calories from fat [Protein 20%, Fat 60%, Carbohydrate 20%, and Energy Density 5.21% Kcal/g] (D12492, Research Diet). After 6 weeks on RCD and HFD, mice were sacrificed, and tissues and blood samples were collected aseptically. GdCl_3_ was dissolved in 0.9% saline and administered to HFD mice at a dose of 50 mg/kg body weight *via* the tail vein, with five doses administered every 3 days. 1 μg of rVim was dissolved in PBS and given to mice *via* tail vein injection, with five doses given every other day. Mice were then sacrificed, and tissues and blood were collected.

### ipGTT and ipITT

ipGTT and ipITT were done as described previously (30, 31). In brief, for the ipGTT, Mice were fasted for 6 h and fed 2 g/kg of glucose, whereas for the ITT, 0.5 IU/kg of body weight insulin (Actrapid, Novo Nordisk, India) was intraperitoneally injected after 6 h of fasting, with free access to water. Blood glucose levels were detected at 15, 30, 60, 90, and 120 min after injection from the tail-tip cut with a glucometer (Accu-Check Aviva).

### Human patients

The study was approved by the human ethics committee of the IIT Mandi. 18 biopsy-proven MAFLD patients and 21 individuals without MAFLD (healthy controls) were recruited for the study. MAFLD patients were diagnosed with histopathological parameters, including steatosis, hepatocellular ballooning, lobular inflammation, and stage of fibrosis. Fasting blood samples were collected from both healthy controls and MAFLD patients. The bio-clinical parameters, including plasma glucose, insulin, total serum cholesterol, TG, aspartate aminotransferase, and alanine aminotransferase, were measured according to the manufacturer's protocol (32).

### Utilization of an online RNA sequencing dataset

RNA sequencing data analysis was performed using a publicly available dataset from the Gene Expression Omnibus database, Accession no. GSE135251. This dataset reports a gene expression analysis of 216 snap-frozen liver biopsies, comprising 206 MAFLD cases with different fibrosis stages and 10 controls (Data ref: Govaere *et al.*, 2020) (33, 34).

### Cell lines and culture treatment

AML-12 cell line was cultured in Dulbecco’s modified Eagle’s medium F-12 (1:1), containing 10% fetal bovine serum, 5 mg/ml insulin, 5 μg/ml transferrin, 5 ng/ml selenium, 40 ng/ml dexamethasone, 100 U/ml penicillin, 100 μg/ml streptomycin, and 2 mmol/L glutamine. Human hepatocellular carcinoma (HepG2 and HEK293T cell lines were cultured in Dulbecco’s modified Eagle’s medium high glucose (4.5 g/L) media supplemented with 10% fetal bovine serum and 1% penicillin-streptomycin and maintained at 37 °C in 5% CO2 incubator. For insulin treatment in KCs, 100 nM insulin was given for 24 h in the presence and absence of STK38 or Vimentin, and conditioned media were collected and used for treatment. For IGF-1R inhibition, HepG2 cells were treated with 700 nM AG1024 for 48 h rVim (100 ng) and AICAR (100 μM) were given for 16 hrs. KCM treatment was given for the indicated time in a 1:100 dilution for the stated period.

### Primary hepatocyte isolation and culture

8-10-week-old mice were used for primary hepatocyte culture according to the previously described protocol (32). In brief, mice were anaesthetized, and after a U-shape incision, the liver was perfused *via* the vena cava with a 30 ml Ca2^+^-free Hank’s balanced salt solution (HBSS) buffer (pH 7.4) containing 1 mM EDTA at a speed of 3 ml/min. Once the liver turned pale, HBSS with Ca^2+^ containing 0.5 mg/ml collagenase was perfused into the liver. Upon observing the cracks on the liver, perfusion was stopped immediately, and the liver was excised into ice-cold HBSS (with Ca^2+)^. Cells from digested livers were teased and filtered through a 100 μm cell strainer and centrifuged at 20 × *g* for 3 min at 4 °C. The pellets were washed with FBS-free William's-E media twice. The cells were cultured in Hepatozyme supplemented with L-glutamine and 1% penicillin-streptomycin on collagen-coated plates for 4 to 6 h. The media was then replaced with complete Hepatozyme supplemented with L-glutamine and 1% penicillin-streptomycin.

### KC isolation and culture

In brief, the portal vein was cannulated, and the liver was perfused with Hank's balanced salt solution without Ca^2+^ or Mg^2+^ (HBSS) for 10 min at 3 ml/min at 30 °C. The liver was then perfused with 0.5 mg/ml collagenase (Sigma-Aldrich; 20 ml at 3 ml/min at 30 °C), softening the parenchyma beneath the capsule. The liver was decapsulated in ice-cold RPMI media and filtered with a 100 μm strainer. The soup was centrifuged at 20 × *g* for 3 min, and pellets of hepatocytes were removed. The supernatant was collected and centrifuged at 250 × *g* for 5 min. The pellet was washed with RBC lysis buffer and centrifuged at 250 × *g* for 3 min. Next, the pellet was washed with serum-free RPMI media and spun at 250 × *g* for 2 min. The pellet was resuspended in RPMI media containing 10% FBS and incubated for 8 h. Cells were washed with PBS, and fresh media was added.

### Preparation of lentivirus

For STK38 knockdown, 293T cells were transfected with pLKO.1 vector containing the shRNA sequence (targeting GCCATACCTTCGTACATGAAA; TRCN0000022864, Sigma-Aldrich) for mouse STK38, along with two packaging vectors, psPAX2 and pMD2.G (ADDGENE). The lentiviral particles were thus generated, purified, and concentrated using an Amicon Ultra-15 centrifugal filter (UFC905008), and approximately 10^12^ pfu/mice were administered *via* tail vein injection. For vimentin knockdown, 293T cells were similarly transfected with the pLKO.1 vector encoding a vimentin-targeting shRNA (GTGGAATCCTTGCAGGAAGAA; Sigma-Aldrich), along with psPAX2 and pMD2.G. An empty pLKO.1 vector (SHC001, Sigma-Aldrich) was used as a control in both experiments. Lentiviral particles were generated, purified, and concentrated using an Amicon Ultra-15 centrifugal filter. For *in vitro* treatment, primary hepatocytes were transduced with STK38- or vimentin-targeting lentiviral particles at an MOI of 6 to 8 in the presence of 8 μg/ml polybrene. After 8 h, the medium was replaced with fresh growth medium and further incubated for total 48 h.

### Western blot analysis

After treatment, cells were lysed in radioimmunoprecipitation assay buffer containing 1 x protease and phosphatase inhibitors and incubated on ice for 30 min with occasional vortexing. Debris was pelleted by centrifuging at 15,000 rpm for 15 min. Protein concentration was determined by the bicinchoninic acid assay reagent as described by the manufacturer’s manual (Thermo Fisher Scientific-23227). Protein was loaded on SDS-PAGE and electroblotted onto polyvinlyidene floride membranes. The membrane was incubated in a 5% nonfat dry milk-blocking solution for 1 h at RT and probed against primary antibody (1:2000 diluted in tris-buffered saline with Tween-20 (TBST)). After washing with TBST 3× for 10 min, the membrane was incubated with horseradish peroxidase-conjugated IgG secondary antibody for 2 h and visualized by chemiluminescence. For mouse plasma Western blotting, plasma samples were diluted 1:1 with PBS, and equal amounts of protein were subjected to SDS–PAGE followed by transfer onto polyvinlyidene floride membranes. Membranes were blocked with 5% nonfat dry milk for 1 h at RT and incubated overnight at 4 °C with anti-vimentin antibody (1:1000 dilution). After TBST washes, membranes were incubated with horseradish peroxidase-conjugated secondary antibodies and visualized by chemiluminescence.

### Immunocytochemistry

Cells were seeded on coverslips precoated with poly-L-lysine. After 16 h of KCM treatment, cells were washed with PBS and fixed with 4% formaldehyde at RT. Further, cells were washed with PBST (0.05% Tween 20 in PBS). Cells were blocked using a 2% FBS blocking buffer prepared in PBS-T and allowed to shake moderately for 1 h at RT. Cells were incubated with primary antibody (1:300 dilution) overnight at 4 °C. After washing with PBST (0.05% Tween-20), cells were incubated with Alexa-Fluor Secondary antibodies at a 1:1000 dilution for 2 h. Cells were washed three times with PBST and mounted with 4',6-diamidino-2-phenylindole mounting media (company). The images were recorded using confocal (Nikon) microscopy.

### Nuclear cytosolic fractionation

Following the manufacturer's protocol, LKB1 nuclear and cytosolic expression was confirmed *via* subcellular fractionation of AML12 cells treated with AICAR (100 μM) and r-Vimentin (100 ng/ml) for 16 h. Briefly, cells were homogenized using the NE-PER Nuclear and Cytoplasmic Extraction Kit (Thermo Fisher Scientific). The nuclear and cytoplasmic fractions were then analyzed using western blotting.

### Bodipy staining

Bodipy staining was done as described previously. In brief, Cells were plated on coverslips, and after 24 h of 100 ng/ml r-Vim treatment, cells were fixed with 4% formaldehyde, washed three times with PBS, stained with 2 μM BODIPY 493/503 (Invitrogen), and incubated at 37 °C for 30 min. Cells were washed 3× with PBS and mounted with 4',6-diamidino-2-phenylindole mounting media. Lipid droplets were analyzed using confocal microscopy (Nikon). BODIPY fluorescence intensity was measured as integrated density by applying a uniform threshold using ImageJ, normalized to cell number, and expressed relative to the control.

### ORO staining

Oil Red O staining of liver sections was performed to evaluate hepatic lipid accumulation. Mouse liver tissues were fixed in 4% paraformaldehyde, cryoprotected in 30% sucrose, embedded in OCT, and sectioned at 5 μm thickness. Slides were then stained with freshly prepared and filtered Oil Red O working solution for 10 min at room temperature. Excess stain was removed by washing with 60% isopropanol followed by distilled water and mounted with mounting medium, and imaged using a light microscope. Oil Red O positive lipid area was quantified using ImageJ by applying a uniform threshold across all images and calculating the percentage of positive area relative to the total field.

### siRNA transfection

HepG2 cells were seeded in a 6-well plate and transfected with 30 nM scramble siRNA and siRNA targeting LKB1 (Silencer s13579) and CAMKK (Silencer s20925) using Lipofectamine RNAiMAX (Invitrogen # 13778150). The reduced serum medium was replaced by complete media after 3 h of siRNA transfection. After 32 h of transfection, the cells were treated with the respective KCM and incubated for 16 h. The cells were lysed and processed for Western blot analysis.

### Statistical analysis

Data were statistically evaluated and plotted using GraphPad Prism. All the data presented is as mean ± SD. To identify statistically significant differences between two groups we used unpaired Student’s *t* test. To compare three or more groups, we used one-way ANOVA followed by post-Bonferroni’s multiple comparisons whenever required. In all cases, the level for statistical significance was 0.05. *p* values less than 0.05, 0.01, 0.001, and 0.0001 are symbolized as ∗/#, ∗∗/##, ∗∗∗/###, and ∗∗∗∗/#### respectively.

## Data availability

This article contains supporting information. All relevant data are within the article and its supporting information files. The raw data can be shared upon request.

## Supporting information

This article contains supporting information.

## Conflict of interest

The authors declare that they have no conflicts of interest with the contents of this article.
