# Supplementary material for: Intermediate filament protein vimentin propels intrahepatic lipid accumulation in insulin-resistant mice
Source: J Biol Chem. 2026 Jun 19;302(8):113265. doi: 10.1016/j.jbc.2026.113265 (PMC13400344; doi:10.1016/j.jbc.2026.113265)
Supplement: Supporting Materials [file mmc1.pdf]

Supporting Materials

Intermediate filament protein Vimentin propels intrahepatic lipid accumulation in insulin-resistant mice

Priya Rawat [1], Shilpa Thakur[1], Kajal Jaswal[1], Aniket Sen[1], Agnimitra Biswal[2], Budheswar Dehury[3,] Prosenjit Mondal[2*]

[1] School of Biosciences and Bioengineering, Indian Institute of Technology Mandi, Mandi-175005, H.P, India. [2] Department of Biological Sciences, Indian Institute of Science Education and Research Berhampur, Berhampur, India. [3] Department of Bioinformatics, Manipal School of Life Sciences, Manipal Academy of Higher Education, Manipal, KA, India.

[*]Corresponding author

Prosenjit Mondal
Department of Biological Sciences,
IISER Berhampur, Berhampur, India
E-mail: pmondal@iiserbpr.ac.in
Phone No.+91-8894296497

Running Title: **Vimentin propels intrahepatic lipid accumulation**

Materials and methods

Secretome analysis from Kupffer cells

Protein samples (n=3 biological replicates per group) were reduced with 5 mM TCEP, alkylated with 50 mM iodoacetamide, and digested with trypsin at a 1:50 (trypsin:lysate) ratio for 16 h at 37 °C. The resulting peptides were desalted using a C18 silica cartridge, dried in a SpeedVac, and resuspended in buffer A (2% acetonitrile, 0.1% formic acid). Approximately 1 µg of peptides was loaded onto a 15 cm C18 column (3.0 µm Acclaim PepMap, Thermo Fisher Scientific) and separated using a 0–40% gradient of buffer B (80% acetonitrile, 0.1% formic acid) at a flow rate of 500 nL/min over 110 min on an Easy-nLC 1000 system coupled to an Orbitrap Exploris mass spectrometer. MS1 spectra were acquired in the Orbitrap at a resolution of 60,000 over a mass range of 375–1500 m/z, followed by data-dependent MS2 acquisition of the top 20 precursor ions at a resolution of 15,000 with dynamic exclusion enabled for 30 s. All samples were processed and RAW files generated were analyzed with Proteome Discoverer (v2.5) against the Uniprot Mus Musculus database and false discovery rate were set to 0.01 FDR. Statistical significance between experimental groups was determined using a two-tailed Student's t-test.

Immunohistochemistry

Bouin's fixed liver tissue were embedded in paraffin and 5µM sections were made, deparaffinized, rehydrated and washed with 1x PBS. Antigen retrieval was performed by microwaving slide for 15 min with sodium citrate buffer (ph 6). Slides were washed and permeabilised with 0.1% triton-X in PBS. Tissue was blocked with 2% Neonatal horse serum for 1 hour and incubated with 1:300 dilution F4/80 (CST) overnight at 4 degrees. Next day, Slides were washed with PBST and incubated with 1:1000 dilution secondary antibody (Alexa-647) for 2hrs and mounted with DAPI mounting media. Imaging was done using confocal microscope (Nikon).

Protein-protein docking of Vimentin with IGF1R using HADDOCK

HADDOCK, protein-protein docking tool that facilitates flexible docking analysis of proteins was used for understanding the molecular recognition of vimentin with full length IGF1R. Earlier studies have shown that vimentin C-terminal end domain (Val330-Glu40) serves as the binding site of IGF1R (Shigyo et al., 2015).[1] Therefore, the experimental structure of vimentin C-terminal domain (PDB: 1GK4-D chain) and full length cryo-EM structure of IGF1R (Li et al., 2019, 6PYH-A chain) was used for knowledge-based docking protocol deployed in HADDOCK.[2,3] The docking process in HADDOCK comprising of initial random orientation of atoms, followed by semiflexible refinement, and concluding with short flexible refinement, which resulted in ten clusters for IGF1R-Vimentin complexes. The optimal structure was selected based on the HADDOCK score and Z-value was employed to identify amino acids involved in hydrogen bonds, non-bonded interactions, and salt bridges using PyMOL and BIOVIA Discovery Studio Visualizer. For a detailed cross-

comparison analysis of the interaction between Vimentin and IGF1 with full length IGF1R, we optimized the IGF1R-IGF1 complex (PDB: 6PYH) using protein-preparation wizard of Maestro and visualized the non-bonded contacts in BIOVIA Discovery Studio Visualizer.

References:

1. Shigyo, M., Kuboyama, T., Sawai, Y., Tada-Umezaki, M. & Tohda, C. Extracellular vimentin interacts with insulin-like growth factor 1 receptor to promote axonal growth. *Sci Rep* **5**, 12055 (2015).
2. Li, J., Choi, E., Yu, H. & Bai, X. Structural basis of the activation of type 1 insulin-like growth factor receptor. *Nat Commun* **10**, 4567 (2019).
3. van Zundert, G. C. P. *et al.* The HADDOCK2.2 Web Server: User-Friendly Integrative Modeling of Biomolecular Complexes. *J Mol Biol* **428**, 720–725 (2016).

Supporting Materials

Table S1. List of secretory proteins found abundant in insulin-treated Kupffer cells conditioned media.

S.no.	Protein FDR Confidence: Combined	Accession	Description	pValue(unpaired t test)	FC	Coverage [%]	# Peptides	# PSMs	# Unique Peptides
1	High	Q5FW60	Major urinary protein 20 OS=Mus musculus OX=10090 GN=Mup20 PE=1 SV=1	0.0378	1.12409	47	8	66	4
2	High	P12710	Fatty acid-binding protein, liver OS=Mus musculus OX=10090 GN=Fabp1 PE=1 SV=2	0.046	1.07383	43	6	94	6
3	High	Q4FZE8	Major urinary protein 1 OS=Mus musculus OX=10090 GN=Mup22 PE=2 SV=1	0.0312	1.0937	47	7	70	1
4	High	P20152	Vimentin OS=Mus musculus OX=10090 GN=Vim PE=1 SV=3	0.0087	1.08762	32	14	80	13
5	High	E9Q1Y3	Apolipoprotein B (Fragment) OS=Mus musculus OX=10090 GN=Apob PE=1 SV=1	0.02	1.06571	3	9	75	9
6	High	Q03265	ATP synthase subunit alpha, mitochondrial OS=Mus musculus OX=10090 GN=Atp5f1a PE=1 SV=1	0.056	1.1419	24	10	35	10
7	High	P08228	Superoxide dismutase [Cu-Zn] OS=Mus musculus OX=10090 GN=Sod1 PE=1 SV=2	0.0678	1.0799	12	2	17	2
8	High	P24270	Catalase OS=Mus musculus OX=10090 GN=Cat PE=1 SV=4	0.0542	1.05346	43	15	82	15
9	High	A0A087WPF8	Abhydrolase domain containing 14b (Fragment) OS=Mus musculus OX=10090 GN=Abhd14b PE=1 SV=1	0.052	1.53369	6	1	1	1
10	High	A0A087WS56	Fibronectin OS=Mus musculus OX=10090 GN=Fn1 PE=1 SV=1	0.035	1.04489	12	20	202	20

Table S2. Baseline characteristics of the study individuals

Variable	Healthy individuals	Patients with MAFLD/MASH
Sample size	21	18
Age (Years)	39.09±3.176	39.167±9.84
Sex: M/F	13/8	9/18
BMI	25.7±3.462	27.14±4.104
TG	147.429±69.652	160.25±50.71
TC	179.045±33.525	187.94±51.34
FBG	90.990±9.098	115.44±61.989
ALT	29.429±24.357	57±58.79
AST	23.286±6.993	46.44±33.393
Hba1c	5.596±0.455	6.11±1.58

Table S3. Intermolecular contact analysis of top ranked complex of IGF1R (Mouse which shares same topology that of human) bound to Vimentin (Val330-Glu407) obtained from HADDOCK (A: IGF1R and B: Vimentin).

Interaction pairs	Distance	Type	Category
A:LYS115:HZ1 - B:ASP331:OD1	1.58	Hydrogen Bond;Electrostatic	Salt Bridge;Attractive Charge
A:ARG690:HH12 - B:GLU368:OE1	2.79	Hydrogen Bond;Electrostatic	Salt Bridge;Attractive Charge
A:ARG690:HH22 - B:GLU368:OE1	1.65	Hydrogen Bond;Electrostatic	Salt Bridge;Attractive Charge
A:ARG690:NH1 - B:GLU368:OE2	4.89	Electrostatic	Attractive Charge
A:CYS671:HN - B:GLU346:OE1	1.80	Hydrogen Bond	Conventional Hydrogen Bond
B:GLN359:HE21 - A:ALA678:O	2.35	Hydrogen Bond	Conventional Hydrogen Bond
B:GLN359:HE21 - A:GLU679:OE1	2.08	Hydrogen Bond	Conventional Hydrogen Bond
B:GLN366:HE21 - A:GLU685:OE2	1.96	Hydrogen Bond	Conventional Hydrogen Bond
B:GLN366:HE22 - A:GLU685:OE1	2.93	Hydrogen Bond	Conventional Hydrogen Bond
B:GLN359:CA - A:GLU679:OE2	3.17	Hydrogen Bond	Carbon Hydrogen Bond
A:ILE701:CD1 - B:HIS379	3.74	Hydrophobic	Pi-Sigma
B:MET376:CE - A:PHE696	3.61	Hydrophobic	Pi-Sigma
B:MET372:SD - A:PHE693	5.63	Other	Pi-Sulfur
A:ALA672 - B:MET347	3.67	Hydrophobic	Alkyl
A:ALA682 - B:ILE362	4.43	Hydrophobic	Alkyl
A:ARG690 - B:ILE369	4.92	Hydrophobic	Alkyl
A:LEU697 - B:MET376	4.55	Hydrophobic	Alkyl
A:PRO706 - B:LYS390	5.48	Hydrophobic	Alkyl
A:TYR138 - B:ALA332	5.13	Hydrophobic	Pi-Alkyl
A:PHE693 - B:ILE369	5.03	Hydrophobic	Pi-Alkyl

A:PHE693 - B:LYS373	4.68	Hydrophobic	Pi-Alkyl
B:PHE351 - A:PRO674	4.66	Hydrophobic	Pi-Alkyl
B:TYR383 - A:VAL703	4.34	Hydrophobic	Pi-Alkyl

Table S4. Intermolecular contact analysis of optimized full-length IGF1R bound to IGF1 using the experimental cryo-EM structure (PDB ID: 6PYH, A: IGF1R and B: IGF1)

Interaction pairs	Distance	Type	Category
A:ARG484:HH12 - B:GLU9:OE2	3.08	Hydrogen Bond; Electrostatic	Salt Bridge;Attractive Charge
A:ARG484:HH22 - B:GLU9:OE1	2.72	Hydrogen Bond;Electrostatic	Salt Bridge;Attractive Charge
A:ARG705:HH12 - B:GLU58:OE1	2.81	Hydrogen Bond;Electrostatic	Salt Bridge;Attractive Charge
A:ARG705:HH12 - B:GLU58:OE2	2.69	Hydrogen Bond;Electrostatic	Salt Bridge;Attractive Charge
A:ARG705:HH22 - B:GLU58:OE1	1.42	Hydrogen Bond;Electrostatic	Salt Bridge;Attractive Charge
A:ARG705:NH2 - B:PRO63:O	4.99	Electrostatic	Attractive Charge
A:LYS691:HZ1 - B:CYS6:SG	2.60	Hydrogen Bond	Conventional Hydrogen Bond
A:ARG705:HE - B:CYS61:O	1.95	Hydrogen Bond	Conventional Hydrogen Bond
A:ARG705:HH21 - B:CYS61:O	1.94	Hydrogen Bond	Conventional Hydrogen Bond
A:ARG705:H - B:MET59:O	1.63	Hydrogen Bond	Conventional Hydrogen Bond
B:THR4:HG1 - A:PRO486:O	2.60	Hydrogen Bond	Conventional Hydrogen Bond
A:PRO704:HA - B:MET59:O	2.25	Hydrogen Bond	Carbon Hydrogen Bond
B:GLY42:HA2 - A:ASN699:OD1	2.03	Hydrogen Bond	Carbon Hydrogen Bond
B:GLU9:OE1 - A:TYR488	3.27	Electrostatic	Pi-Anion
B:TYR24 - A:VAL703	5.47	Hydrophobic	Pi-Alkyl
B:TYR24 - A:ARG705	4.32	Hydrophobic	Pi-Alkyl
B:TYR60 - A:PRO704	3.81	Hydrophobic	Pi-Alkyl

Supporting Materials

Supplementary Information

S1.a

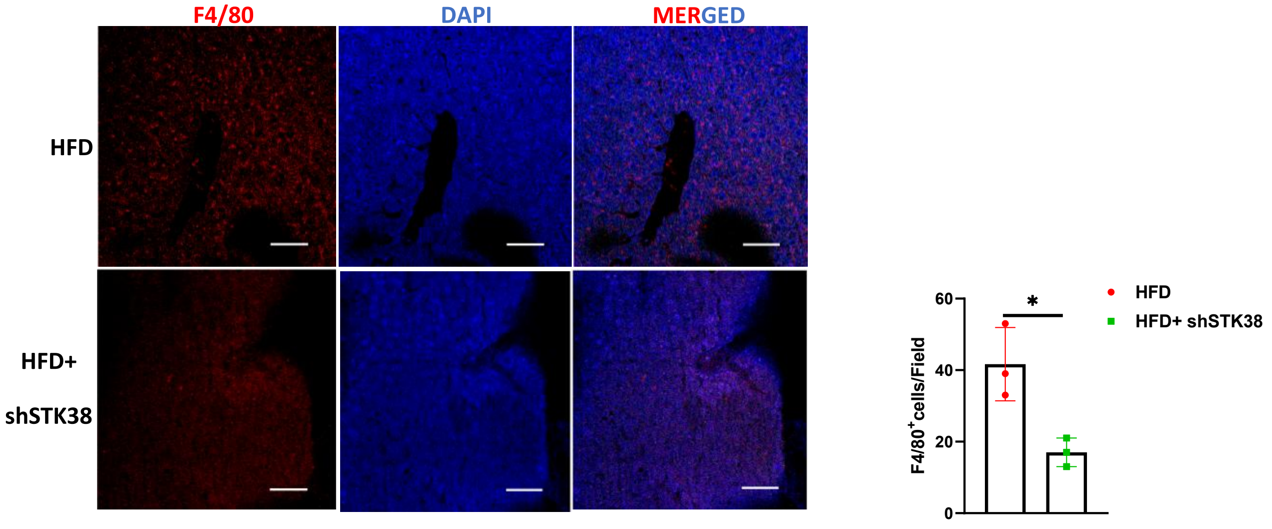

S1.b

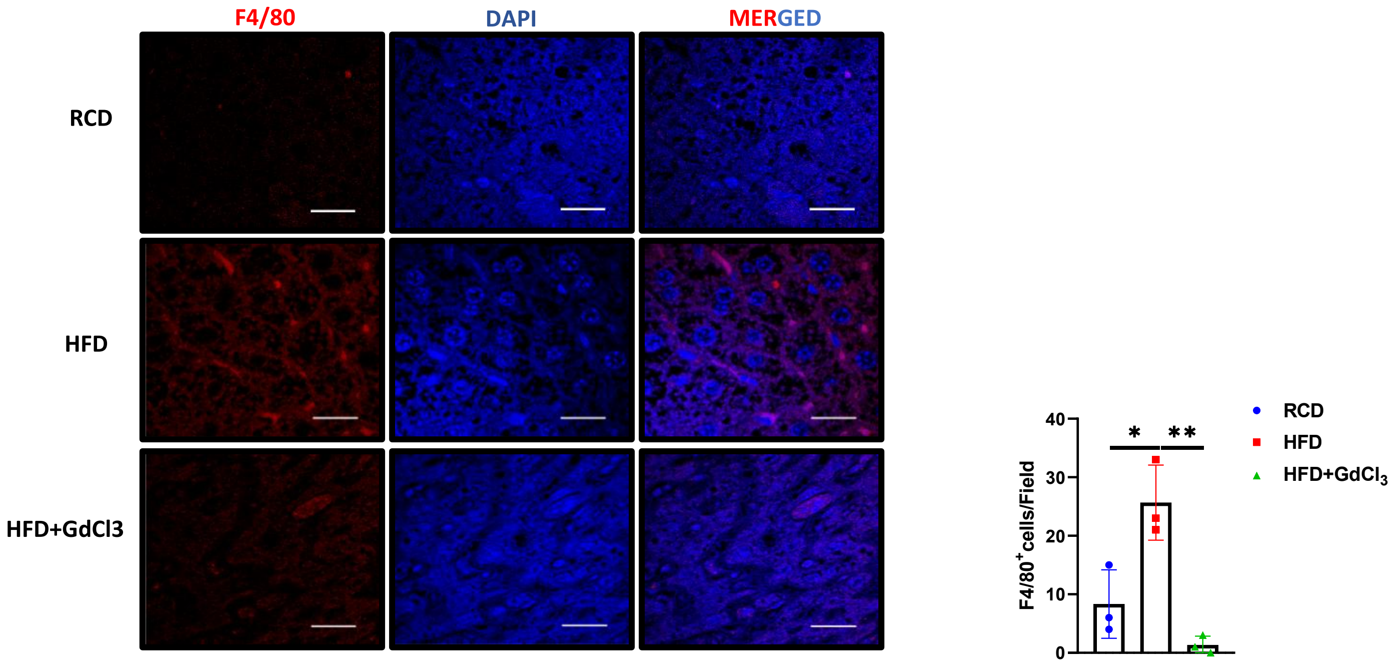

S1.c

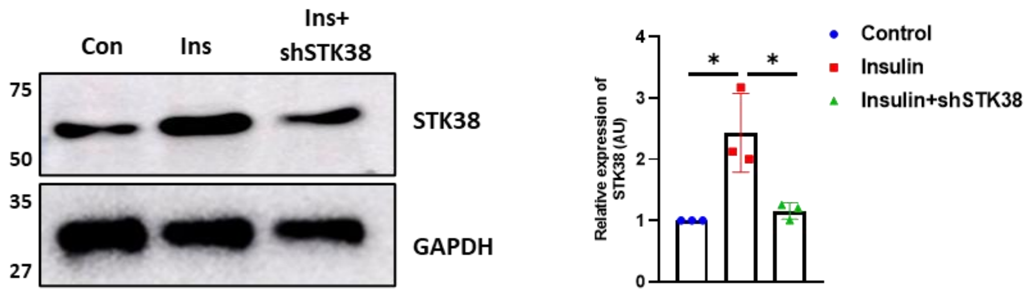

Supporting Materials

S2.a

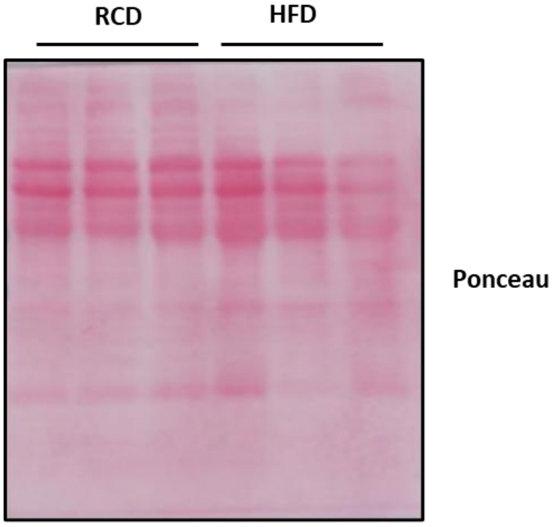

S2.b

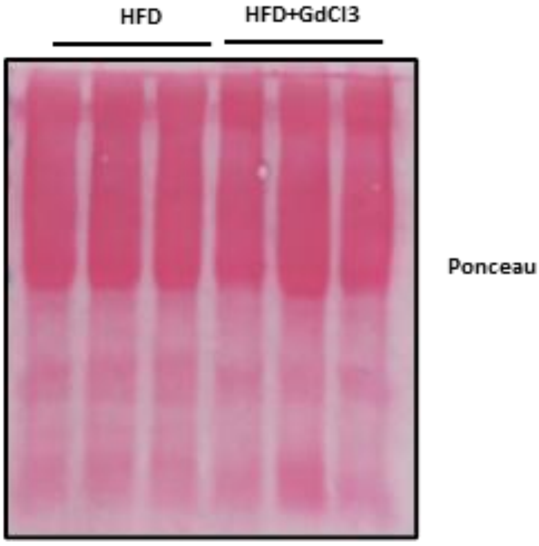

S2.c

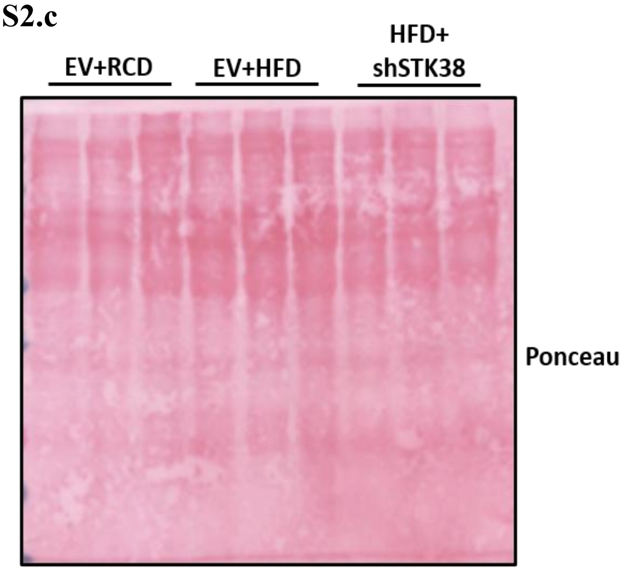

S2.d

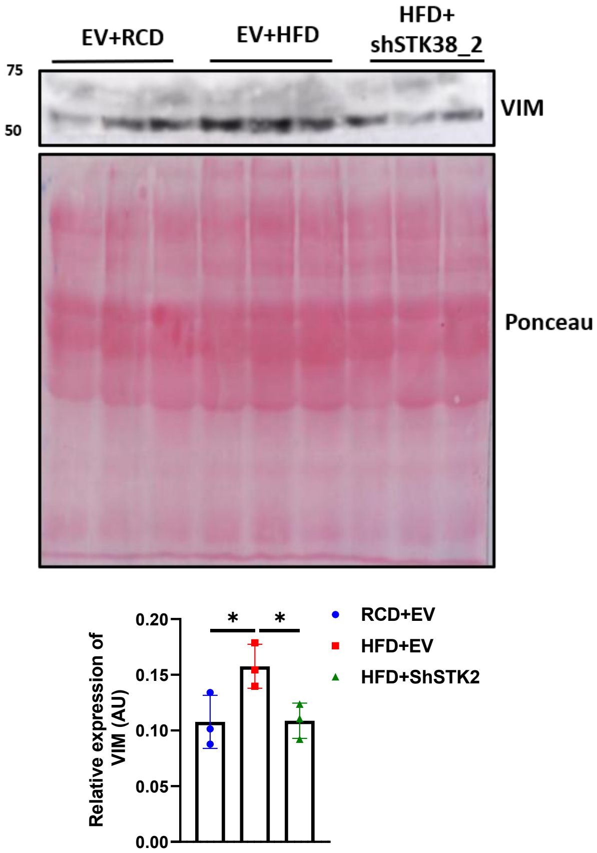

S2.e

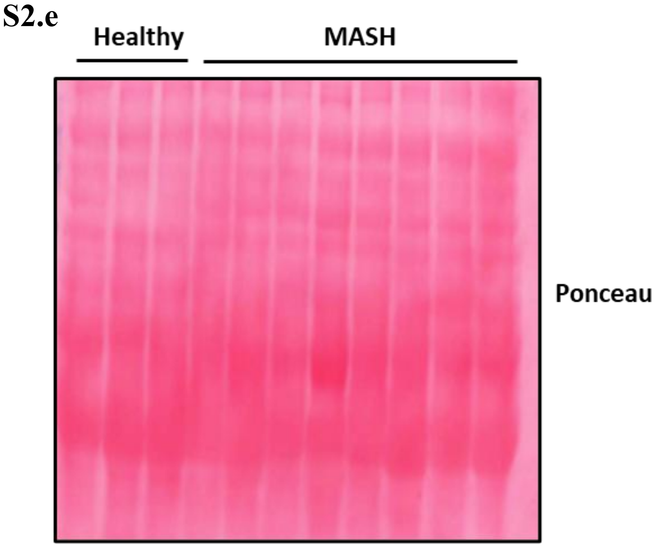

Supporting Materials

S2.f

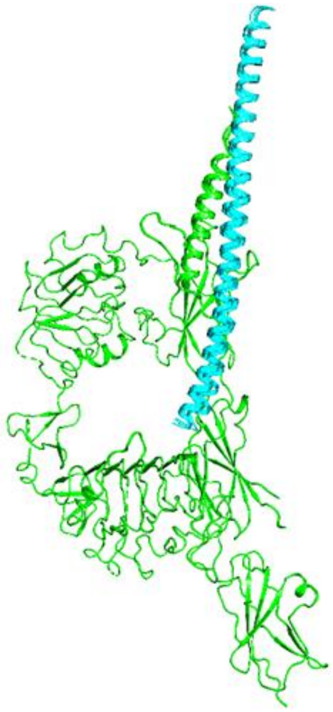

S2.g

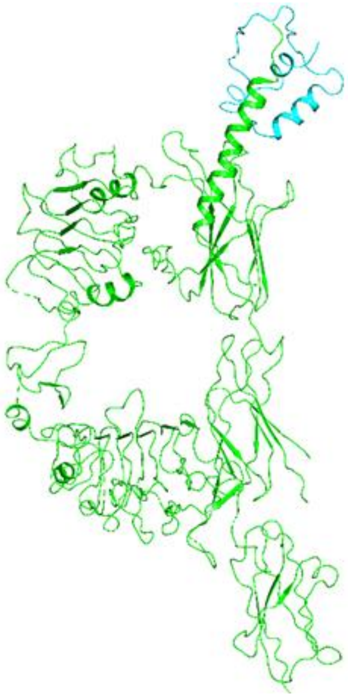

S2.h

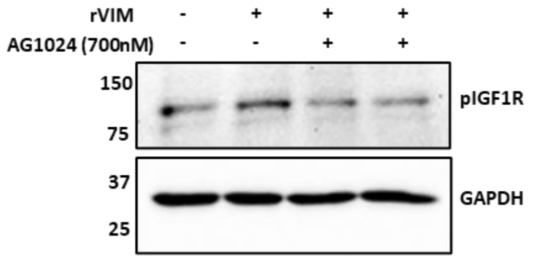

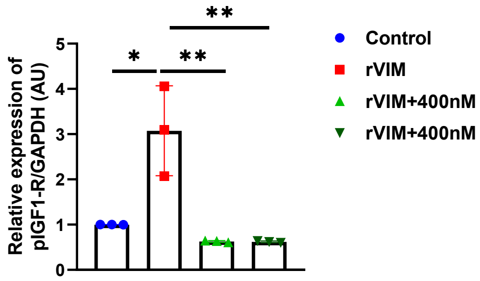

S3.a

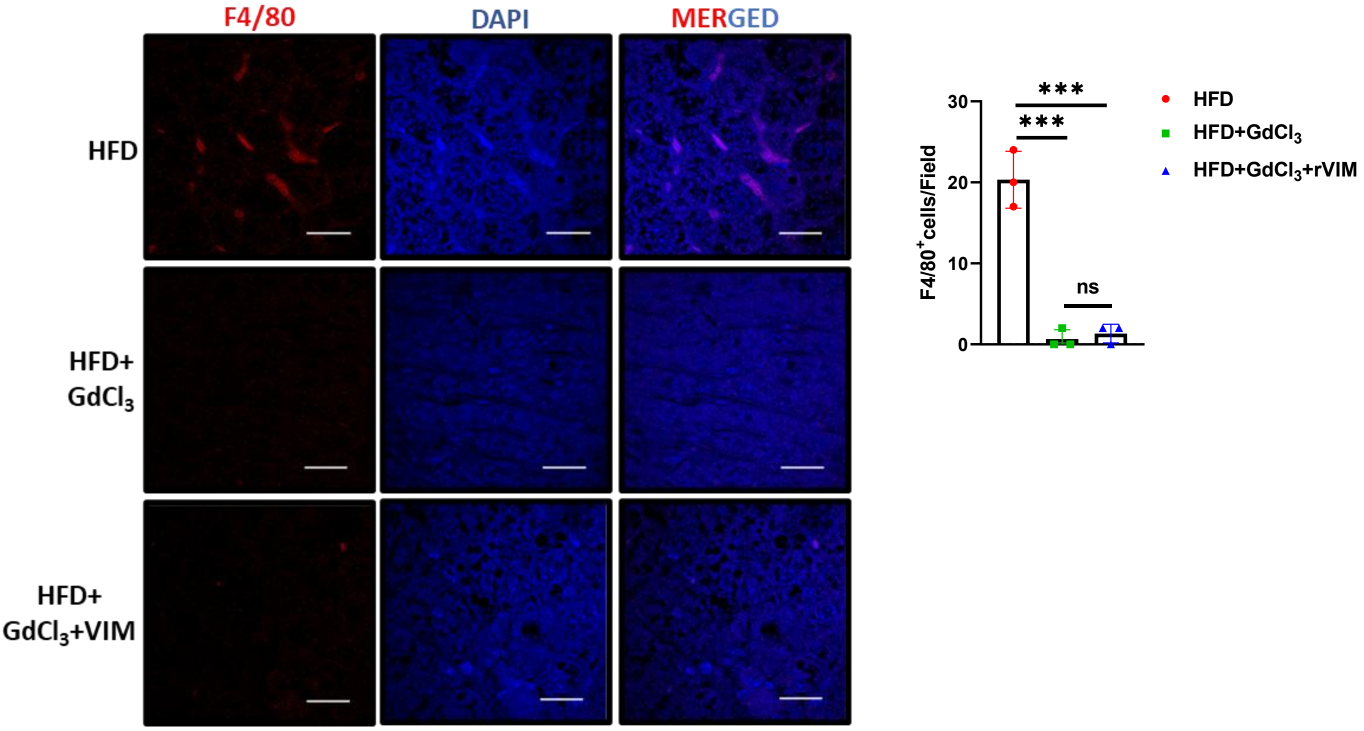

S3.b

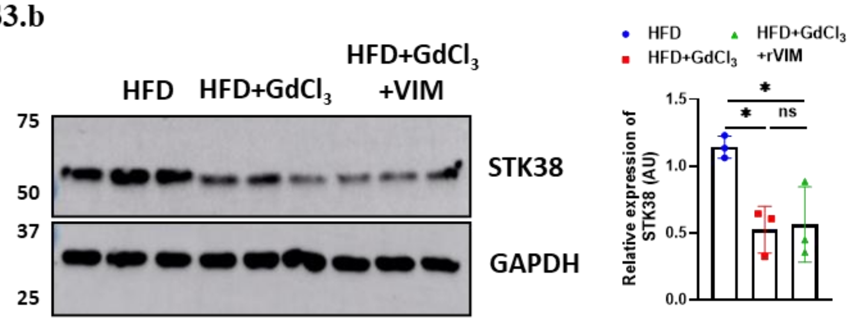

Supplementary figures legends:

S.Fig.1. Enhanced expression of STK38 in Kupffer cells. (a) F4/80 staining in the liver of HFD and HFD+shSTK38 mice. Red fluorescence F4/80 and blue fluorescence DAPI. Scale 20µm**.** Quantification of F4/80-positive cells was performed by counting the number of F4/80$^+$ cells per field using ImageJ. **(b)** F4/80 staining in the liver of RCD, HFD, and HFD+$GdCl_3$ mice. **Red** fluorescence F4/80 and blue fluorescence DAPI. Scale 20µm. Quantification of F4/80-positive cells was performed by counting the number of F4/80$^+$ cells per field using ImageJ. **(c)** Qualitative and quantitative expression of STK38 normalized by GAPDH in Kupffer Cells after treatment with 100 nM insulin with or without shSTK38. Data is represented as Mean±SD. n represents biological replicates. Comparisons between samples in panel S1.a were conducted using an unpaired Student's t-test. Significance levels are denoted as $*P < 0.05$. Comparisons across the treatments (S1.b&c) were performed using one-way ANOVA followed by Bonferroni's multiple comparisons test. Statistical significance is indicated as: $*P < 0.05$, $**P < 0.01$.

S.Fig.2. Vimentin and IGF1 bind to different, distinct regions on IGF1R. (a) Ponceau S staining of RCD and HFD mice serum proteins used for normalization. **(b)** Ponceau S staining of HFD and HFD+$GdCl_3$ mice serum proteins used for normalization. **(c)** Ponceau S staining of RCD + EV (empty vector control), HFD + EV, and HFD + shSTK38 mice serum proteins used for normalization. **(d)** Qualitative and quantitative representation of the Vimentin protein level normalized to total protein in the serum of RCD + EV, HFD + EV, and HFD + shSTK38_2 mice (n = 3 each), where shSTK38_2 represents the second shRNA construct targeting a different site of the *STK38* gene. **(e)** Ponceau S staining of Healthy and MASH Human serum proteins used for normalization. **(f)** Mode of binding of Vimentin (cyan) with full-length IGF1R (green) obtained from protein-protein docking using HADDOCK. **(g)** Optimized complex showing the mode of binding of IGF1 (cyan) with full-length IGF1R (green). **(h)** Phosphorylation of IGF1-R normalized by GAPDH in HepG2 cells after being treated with rVim for 15 mins, where AG1024 was pre-treated for 48 hours before the treatment of rVim, and analyzed by western blot and quantified using ImageJ. Data is represented as Mean±SD. n represents biological replicates. Comparisons between samples in panel S2.g were performed using one-way ANOVA followed by Bonferroni's multiple comparisons test. Statistical significance is indicated as: $^{*}P < 0.05$, $^{**}P < 0.01$.

S.Fig.3. Vimentin counteracts the positive effect of $GdCl_3$. (a) F4/80 staining in the liver of HFD, HFD+$GdCl_3$, and HFD+$GdCl_3$+rVim treated mice. **Red** fluorescence F4/80 and blue fluorescence DAPI. Scale 20μm. Quantification of F4/80-positive cells was performed by counting the number of F4/80^{+} cells per field using ImageJ. **(b)** Qualitative and quantitative expression of STK38 normalized by GAPDH in the liver of HFD, HFD+$GdCl_3$, and HFD+$GdCl_3$+rVim. Data is represented as Mean±SD. n represents biological replicates. Comparisons across the treatments (S3.a&b) were performed using one-way ANOVA followed by Bonferroni's multiple comparisons test. Statistical significance is indicated as: ns: not significant ($p>0.05$), $^{*}P < 0.05$, $^{***}P < 0.001$.

Supporting Materials

List of Proteins identified in Secretome by LC/MS analysis

Protein FDR Confidence: Combined	Accession	pValue	# Peptides	# PSMs	Coverage [%]	# Unique Peptides	CON_1	CON_2	CON_3	INS_1	INS_2	INS_3	Modifications
High	A0A0A0MQM0	0.2191	3	16	30	3	18.344	16.175	17.683	17.603	18.770	19.645	NA
High	A0A087WPF8	0.0521	1	1	6	1	7.643	10.042	12.442	13.003	15.402	17.801	Acetyl [N-Term]
High	A0A1B0GSX0	0.4173	7	40	20	6	20.897	20.621	20.452	20.139	20.410	20.791	NA
High	A0A1C7CYU3	0.4356	3	12	8	3	17.817	18.473	18.278	17.497	17.760	18.424	NA
High	A0A1C7CYV0	0.4176	16	107	33	16	17.606	18.517	19.333	18.959	18.191	21.133	Acetyl [N-Term]
High	A0A6I8MX27	0.8289	12	114	37	11	21.928	21.800	21.345	21.103	20.970	22.602	NA
High	A2A4I9	0.0282	2	16	2	2	21.219	20.871	20.670	21.821	21.642	21.701	NA
High	A2ALB3	0.0013	1	4	1	1	18.303	17.596	18.206	15.266	14.783	15.680	NA
High	A6ZI44	0.5094	9	66	22	8	21.292	21.690	21.090	21.469	21.321	21.759	NA
High	B7FAV1	0.0810	8	43	3	5	23.129	21.535	20.744	19.949	19.537	19.349	NA
High	E9PZF0	0.9784	6	72	45	3	21.148	20.692	20.450	20.146	20.318	21.878	NA
High	E9Q5F6	0.4409	4	38	61	4	21.299	21.488	20.198	21.488	20.880	21.916	NA
High	E9QNY8	0.0290	1	6	0	1	20.803	21.080	20.424	21.940	21.299	21.769	NA
High	G5E850	0.8065	5	10	58	5	20.028	20.470	21.063	19.781	20.527	20.902	NA
High	O08807	0.9110	5	37	20	4	18.449	19.384	17.823	17.317	18.660	20.004	NA
High	O35453	0.6190	1	50	2	1	23.668	23.347	23.053	24.460	24.905	22.188	NA
High	P00329	0.0918	5	28	12	5	20.657	20.493	21.636	22.302	23.037	21.421	NA
High	P00687	0.4125	3	55	7	3	22.106	22.347	22.049	22.136	22.089	21.986	NA
High	P04186	0.0617	8	28	12	8	20.931	20.799	21.188	21.552	21.603	22.351	NA
High	P05213	0.5321	17	192	48	4	22.585	22.487	22.415	22.357	22.424	23.661	NA
High	P05784	0.9946	5	29	16	5	20.804	18.697	19.691	20.062	20.617	18.533	NA
High	P07901	0.4902	19	188	26	10	21.364	21.005	20.887	21.346	20.761	22.271	NA
High	P08071	0.5796	2	49	2	1	22.063	22.556	20.345	21.494	20.072	21.837	NA
High	P08226	0.0933	11	91	38	11	23.657	25.084	23.942	22.880	22.784	23.414	NA
High	P09411	0.6735	11	84	27	7	21.223	21.024	20.702	19.994	20.629	21.617	NA
High	P10126	0.8645	11	149	31	5	23.955	22.699	23.274	22.797	22.938	23.916	NA

Supporting Materials

High	***P10639***	0.0207	2	22	21	2	19.576	19.994	20.126	20.482	21.080	20.800	NA
High	***P10649***	0.2081	8	91	40	8	20.325	20.836	22.330	22.687	22.137	21.897	NA
High	***P11087***	0.1048	6	81	5	6	23.889	23.946	23.930	23.200	23.141	21.946	NA
High	***P11499***	0.6379	24	219	34	13	23.431	22.581	22.823	22.709	22.937	23.852	NA
High	***P11725***	0.5586	6	33	21	6	20.183	19.435	21.194	20.085	21.048	20.826	NA
High	***P11983***	0.1431	4	11	7	4	19.637	20.080	17.153	16.203	17.243	17.328	NA
High	***P14131***	0.5523	1	6	7	1	16.527	16.745	16.632	15.940	16.137	17.077	NA
High	***P14206***	0.3374	3	7	16	3	17.911	17.876	19.270	16.856	16.686	18.795	Acetyl [N-Term]
High	***P16460***	0.2101	9	57	27	9	21.016	21.808	22.384	22.191	22.239	23.850	NA
High	***P17182***	0.4989	14	180	41	13	22.667	22.365	22.684	22.521	22.528	23.394	NA
High	***P17742***	0.7955	8	73	40	8	21.537	21.493	21.622	21.229	21.390	22.341	NA
High	***P17751***	0.6906	9	88	43	9	21.013	20.759	20.853	20.207	20.057	21.654	NA
High	***P18760***	0.6620	7	36	49	6	21.533	21.969	21.784	21.613	21.732	22.294	Acetyl [N-Term]
High	***P19157***	0.2223	7	84	41	7	22.707	22.447	23.460	23.522	23.449	23.207	NA
High	***P19324***	0.1236	2	10	6	2	17.698	18.030	18.366	16.931	16.721	17.880	NA
High	***P20152***	0.0087	14	80	32	13	20.299	19.836	19.411	22.051	21.556	21.160	NA
High	***P26039***	0.2511	25	178	13	21	22.818	22.897	22.692	22.545	22.512	22.842	NA
High	***P26041***	0.3115	16	89	26	10	18.762	19.572	21.094	20.270	20.842	21.064	NA
High	***P30115***	0.0452	5	18	26	5	21.893	21.642	21.425	19.938	19.938	20.985	Acetyl [N-Term]
High	***P40142***	0.3478	7	28	12	7	20.492	20.132	20.124	19.384	19.456	20.540	NA
High	***P43274***	0.0860	6	48	22	6	20.994	22.192	21.574	19.847	19.349	21.102	NA
High	***P49182***	0.2459	2	80	6	2	21.988	22.613	22.372	23.340	23.242	22.184	NA
High	***P50396***	0.1906	5	34	15	3	19.224	18.901	18.685	18.927	19.729	20.058	NA
High	***P50580***	0.2692	4	16	10	4	18.618	17.139	15.816	17.908	18.609	18.699	NA
High	***P52480***	0.6263	15	112	37	15	21.763	21.484	20.869	21.443	21.113	22.232	NA
High	***P58252***	0.3045	21	135	25	21	20.534	20.833	19.947	20.408	20.928	22.243	NA
High	***P60710***	0.1763	23	464	74	8	27.091	27.370	26.787	26.586	26.658	26.968	Acetyl [N-Term]
High	***P60843***	0.4367	14	68	36	14	19.973	19.801	19.810	19.488	20.315	21.283	NA
High	***P61982***	0.2514	9	96	38	4	20.319	20.535	21.165	20.846	20.958	21.748	NA
High	***P62259***	0.1196	11	77	53	9	20.860	19.647	20.100	20.597	21.354	22.240	Acetyl [N-Term]
High	***P62702***	0.5673	6	26	21	6	18.862	19.106	19.211	18.621	17.815	19.652	NA

Supporting Materials

High	***P62806***	0.0267	8	113	52	8	24.450	23.672	24.080	23.137	22.651	23.312	NA
High	***P62897***	0.0298	2	10	18	2	19.854	19.357	18.914	17.823	17.283	18.493	NA
High	***P63017***	0.7543	23	198	38	20	23.284	23.341	22.787	22.604	22.780	23.651	NA
High	***P63038***	0.1013	14	75	32	14	22.321	21.822	22.469	24.268	22.960	22.916	NA
High	***P63101***	0.8707	9	104	41	5	22.601	22.501	22.413	22.284	22.328	23.040	Acetyl [N-Term]
High	***P80314***	0.2587	6	15	13	6	19.333	18.950	18.058	18.959	19.428	19.766	Acetyl [N-Term]
High	***P80315***	0.4211	6	25	15	6	19.142	19.646	17.947	17.091	17.747	19.504	NA
High	***P84244***	0.3044	3	8	22	3	22.757	22.395	22.648	22.674	22.165	22.290	NA
High	***P86048***	0.2213	1	4	6	1	17.394	16.577	16.459	16.032	15.884	16.768	NA
High	***P97290***	0.0460	1	20	1	1	20.660	20.564	21.721	22.709	22.444	22.486	NA
High	***P97351***	0.8135	2	28	7	2	17.221	17.744	17.829	16.536	16.860	18.814	NA
High	***P99024***	0.8315	15	172	44	4	20.020	19.614	18.289	18.890	19.368	20.098	NA
High	***Q05186***	0.4116	4	14	15	4	17.632	17.590	17.413	16.805	17.445	17.617	NA
High	***Q5SX59***	0.2546	2	34	13	1	16.969	17.000	16.251	17.691	16.598	18.391	NA
High	***Q5U405***	0.6230	1	67	1	1	29.676	29.766	29.950	30.706	30.706	28.979	NA
High	***Q60668***	0.5506	2	6	6	2	17.334	17.008	17.727	17.321	17.444	17.802	NA
High	***Q60864***	0.6820	15	51	24	15	17.228	19.107	19.390	18.183	18.486	20.314	NA
High	***Q61171***	0.1348	2	18	9	2	20.585	20.627	20.130	20.100	19.763	20.262	NA
High	***Q61176***	0.4656	8	34	35	8	23.687	23.387	23.364	24.447	24.511	22.900	NA
High	***Q61696***	0.8080	14	141	31	7	19.807	20.020	18.885	19.437	17.769	22.735	NA
High	***Q62000***	0.2556	3	18	9	3	19.915	19.468	19.615	20.739	20.306	19.561	NA
High	***Q63880***	0.2145	6	20	17	6	18.710	18.699	20.676	20.267	20.533	20.777	NA
High	***Q64475***	0.1086	7	105	52	7	24.332	23.811	23.722	23.566	22.266	23.118	NA
High	***Q6GT24***	0.2270	5	28	28	5	19.273	19.524	18.799	19.614	19.492	19.581	NA
High	***Q6IRU2***	0.1822	5	21	16	4	18.646	19.699	19.898	18.539	18.777	18.613	Acetyl [N-Term]
High	***Q792Z1***	0.3433	1	60	4	1	27.141	27.792	27.423	27.636	28.650	27.468	NA
High	***Q8BFU2***	0.0399	5	59	58	2	24.460	24.236	23.139	22.523	21.899	22.670	NA
High	***Q8BSH9***	0.0697	3	22	10	3	18.242	16.866	16.224	15.885	15.052	14.842	NA
High	***Q8BWZ3***	0.0322	1	9	2	1	25.487	25.302	24.405	26.590	26.807	25.983	NA
High	***Q8VCT4***	0.2156	8	43	17	8	21.190	21.492	21.549	19.816	20.610	21.395	NA
High	***Q8VDD5***	0.0004	9	28	7	9	20.674	20.596	20.698	20.153	20.246	20.161	NA

Supporting Materials

High	Q8VHX6	0.1322	4	14	1	1	17.538	16.282	16.977	18.004	17.292	18.079	NA
High	Q921I1	0.0345	18	131	24	16	27.522	27.311	26.419	25.945	25.356	25.722	NA
High	Q921R2	0.1561	2	6	17	2	16.855	17.115	16.292	17.520	16.946	17.397	NA
High	Q93092	0.2880	3	23	9	3	16.998	18.261	17.592	18.114	17.709	18.844	NA
High	Q9CPN9	0.9864	1	10	5	1	22.402	23.318	22.319	21.747	23.568	22.690	NA
High	Q9CQV8	0.8377	8	92	32	2	19.162	18.319	19.701	18.654	18.460	20.594	NA
High	Q9D0R2	0.2166	3	6	4	3	17.673	16.289	16.967	17.608	17.532	17.888	NA
High	Q9ERK4	0.4091	2	6	3	2	22.210	20.987	21.973	21.592	20.901	21.452	NA
Medium	Q8CDI6	0.0287	1	2	1	1	22.197	21.147	20.281	19.412	17.897	16.959	NA
High	A0A075B5Q3	0.3392	1	4	8	1	21.548	22.410	15.676	17.841	17.503	16.401	NA
High	A0A087WPD2	0.0864	1	75	10	1	25.993	25.837	26.275	26.730	26.702	26.190	NA
High	A0A087WS16	0.2883	6	30	2	6	19.303	19.184	19.876	20.721	19.431	19.872	NA
High	A0A087WS56	0.0354	20	202	12	20	24.355	24.768	24.649	26.183	25.619	25.281	NA
High	A0A0A0MQA5	0.9623	14	154	38	1	19.541	20.100	18.232	19.293	19.456	19.212	NA
High	A0A0R4J0I9	0.7417	4	15	1	4	16.629	19.929	18.876	18.108	19.032	17.081	NA
High	A0A0U1RQB4	0.8224	4	14	41	4	22.041	22.361	23.567	22.585	22.636	23.117	NA
High	A0A1D5RLD8	0.7145	12	118	40	12	23.507	22.658	21.887	22.215	21.999	23.144	NA
High	A0A1L1SSH9	0.9573	3	30	7	3	20.503	20.304	19.573	19.810	20.139	20.490	NA
High	A0A1L1SV25	0.6667	12	66	17	5	21.684	20.419	20.106	20.388	20.220	20.851	NA
High	A0A1W2P6G5	0.0959	3	16	23	3	21.106	21.106	19.621	18.814	17.959	14.690	NA
High	A0A2I3BRQ3	0.2638	9	242	10	9	27.278	27.531	25.054	25.997	25.607	24.609	NA
High	A0A338P692	0.1971	2	16	17	2	25.336	25.211	25.738	25.509	25.813	26.314	Acetyl [N-Term]
High	A0A338P731	0.2892	1	4	8	1	20.900	20.574	15.728	17.602	16.463	16.011	NA
High	A1BN54	0.1377	9	62	12	2	17.558	17.819	17.030	16.775	17.110	15.767	NA
High	A2A513	0.1421	5	90	10	4	19.812	19.812	24.585	25.434	26.092	23.327	NA
High	A2A816	0.1502	2	5	19	2	20.814	20.528	16.906	17.925	15.090	16.906	NA
High	A2A998	0.4875	1	6	1	1	14.685	19.410	18.662	19.318	18.358	18.810	NA
High	A2APM3	0.2528	1	24	2	1	19.525	19.494	17.899	19.378	20.597	19.615	NA
High	B0V2N8	0.1728	1	4	9	1	18.740	17.777	16.076	16.815	15.883	13.360	NA
High	B1B0C7	0.6089	2	10	1	2	21.072	20.885	19.586	19.453	20.885	20.166	NA
High	B8JK33	0.2838	3	12	5	3	17.644	17.063	18.469	15.818	16.233	18.054	NA

Supporting Materials

High	***D3YXF5***	0.5140	1	14	2	1	18.165	17.138	18.639	19.631	18.115	17.760	NA
High	***D3YZ61***	0.4613	3	35	10	3	20.191	21.161	20.799	20.504	21.780	21.016	NA
High	***E0CZA1***	0.5446	1	6	5	1	18.871	18.285	14.659	18.985	18.530	17.247	NA
High	***E9PUC5***	0.8880	1	10	2	1	18.368	18.356	19.557	19.366	18.626	18.508	NA
High	***E9PV24***	0.9715	1	14	1	1	16.436	22.630	19.586	19.168	19.673	19.595	NA
High	***E9PWF0***	0.7654	3	34	3	1	17.227	18.566	16.767	17.160	17.976	16.802	NA
High	***E9Q0F0***	0.2117	2	38	1	2	22.026	22.799	23.202	25.207	24.639	22.521	NA
High	***E9Q1V0***	0.9004	1	6	8	1	18.101	17.492	15.221	17.457	15.693	17.244	NA
High	***E9Q1X8***	0.6710	2	3	3	2	17.730	17.167	18.866	16.892	19.469	18.668	NA
High	***E9Q1Y3***	0.0289	9	75	3	9	21.824	22.281	21.254	23.018	23.824	22.812	NA
High	***E9Q5L2***	0.6274	2	78	2	2	25.789	25.791	26.236	26.732	25.945	25.699	NA
High	***E9QPX1***	0.1685	2	12	1	2	19.222	19.378	19.064	20.202	20.242	19.241	NA
High	***F8WJ05***	0.2028	1	14	1	1	17.008	16.969	14.626	19.203	17.638	16.684	NA
High	***G3UY13***	0.1261	2	18	5	2	19.318	19.440	18.973	20.231	19.587	19.527	NA
High	***G3X8Q5***	0.7504	7	52	9	7	20.664	20.966	22.132	21.408	21.194	21.661	NA
High	***H7BX99***	0.8688	6	45	12	6	23.300	23.175	24.241	25.403	23.455	22.377	NA
High	***O08677***	0.9305	2	15	3	2	24.436	24.821	23.307	24.169	24.352	23.906	NA
High	***O08710***	0.6223	2	12	1	2	19.852	20.171	19.922	20.171	19.583	19.873	NA
High	***O35103***	0.0873	1	14	2	1	19.699	19.406	20.515	21.342	21.044	20.304	NA
High	***O55222***	0.7281	1	4	2	1	17.630	16.833	17.492	18.108	15.178	17.570	NA
High	***O88200***	0.3048	2	16	8	2	19.103	19.030	18.169	17.922	18.987	17.178	NA
High	***O88569***	0.7325	6	42	20	5	20.115	19.754	20.027	19.181	18.980	20.981	NA
High	***O88783***	0.2356	11	96	4	11	23.275	23.919	25.795	25.942	25.474	25.330	NA
High	***O88947***	0.5156	2	24	4	2	22.476	22.250	20.838	21.691	21.892	20.572	NA
High	***O89020***	0.8282	2	76	2	2	23.156	23.245	22.382	22.986	23.595	22.495	NA
High	***P01027***	0.3792	16	263	10	16	27.295	27.460	27.336	27.616	27.362	27.397	NA
High	***P01029***	0.6320	6	83	3	6	24.189	24.247	23.773	24.399	24.326	23.841	NA
High	***P02088***	0.2152	2	62	13	1	22.538	22.538	26.438	25.919	26.734	25.748	NA
High	***P02104***	0.6604	3	49	22	2	27.145	27.577	25.546	26.688	26.633	25.968	NA
High	***P02762***	0.4564	7	78	50	1	16.145	18.447	17.922	18.831	17.173	18.690	NA
High	***P04104***	0.1921	5	48	6	3	20.602	22.572	24.289	26.274	25.405	22.798	NA

Supporting Materials

High	*P04939*	0.3120	5	54	24	2	20.086	18.264	21.039	20.572	21.252	20.826	NA
High	*P05202*	0.8364	7	38	16	7	20.367	20.263	21.859	19.922	21.603	21.456	NA
High	*P06684*	0.7445	1	14	1	1	19.258	19.155	19.525	19.796	19.951	17.179	NA
High	*P07724*	0.7552	20	1594	32	20	30.338	30.462	30.016	30.580	30.033	29.964	NA
High	*P07758*	0.7691	5	30	12	1	18.751	20.040	21.265	19.707	18.000	21.231	NA
High	*P08113*	0.4519	13	96	16	11	21.697	21.018	20.953	20.454	24.337	22.024	NA
High	*P08121*	0.4950	3	31	2	3	20.783	20.816	21.323	21.392	21.015	21.005	NA
High	*P08228*	0.0678	2	17	12	2	18.161	18.814	19.802	20.658	20.893	19.767	NA
High	*P08249*	0.1720	7	34	28	7	20.543	21.293	21.104	17.714	17.971	20.924	NA
High	*P09103*	0.5808	9	67	17	9	20.773	20.804	21.597	19.876	20.682	21.580	NA
High	*P09528*	0.1747	3	12	15	3	19.638	18.837	21.467	21.117	22.047	21.443	NA
High	*P0DP27*	0.5371	3	8	20	3	19.641	17.306	17.036	17.478	17.748	16.896	Acetyl [N-Term]
High	*P11672*	0.5892	1	2	6	1	18.017	16.806	19.422	15.026	18.038	18.746	NA
High	*P11679*	0.3183	14	106	25	8	21.647	21.920	23.882	25.972	24.326	21.952	NA
High	*P12710*	0.0469	6	94	43	6	20.419	20.890	21.874	23.153	22.177	22.518	NA
High	*P12960*	0.3441	9	56	10	9	20.533	20.874	19.893	20.605	21.486	20.553	NA
High	*P13020*	0.8881	22	273	32	22	25.607	25.828	25.828	26.337	26.065	25.051	NA
High	*P14152*	0.1285	5	36	18	5	21.485	21.586	21.552	22.250	22.858	21.804	NA
High	*P14211*	0.2060	5	18	11	5	23.324	23.284	21.780	21.687	21.701	22.254	NA
High	*P16015*	0.9061	7	29	31	7	20.250	20.680	22.965	18.551	24.236	21.820	NA
High	*P16301*	0.3302	1	18	2	1	19.715	19.791	19.084	20.476	20.271	19.299	NA
High	*P16627*	0.5426	9	86	19	1	20.498	20.106	18.224	20.782	18.857	15.963	NA
High	*P20029*	0.4438	20	156	32	18	22.212	22.119	22.679	20.804	22.280	22.432	NA
High	*P20918*	0.6094	2	42	2	2	21.278	21.083	19.574	20.498	19.610	20.749	NA
High	*P24270*	0.0543	15	82	43	15	22.079	22.457	22.990	23.023	24.090	24.024	NA
High	*P24369*	0.3530	4	18	19	4	17.322	17.483	20.022	19.465	18.636	19.915	NA
High	*P25688*	0.5637	15	120	45	15	22.654	23.086	24.121	23.073	24.087	23.705	Acetyl [N-Term]
High	*P26443*	0.4705	12	75	26	12	20.948	20.912	22.805	21.289	22.626	22.598	NA
High	*P27773*	0.2319	8	23	18	8	20.036	21.013	20.689	18.521	19.638	20.583	NA
High	*P28481*	0.3853	4	31	2	4	20.794	21.795	19.968	21.198	20.111	17.951	NA
High	*P28665*	0.1558	2	30	2	2	25.643	25.911	26.773	27.010	27.227	26.366	NA

Supporting Materials

High	***P29391***	0.9673	4	20	27	4	18.331	18.313	20.689	18.833	18.356	20.020	NA
High	***P32020***	0.6008	8	38	12	8	17.921	20.131	21.944	14.848	19.087	21.944	NA
High	***P32261***	0.6152	13	243	32	13	26.010	25.936	25.978	26.814	26.438	25.413	NA
High	***P35441***	0.7118	12	197	10	11	24.576	24.836	24.201	24.867	24.931	24.178	NA
High	***P35700***	0.3942	7	56	31	6	22.154	21.741	20.357	22.079	22.040	21.890	NA
High	***P38647***	0.4959	4	13	6	4	17.595	17.086	19.639	14.751	15.761	19.796	NA
High	***P47738***	0.6575	9	36	21	9	17.742	17.762	22.320	15.803	15.877	22.320	NA
High	***P47877***	0.5763	1	8	4	1	19.071	19.683	15.496	20.823	17.707	18.661	NA
High	***P47880***	0.9362	1	20	6	1	20.702	18.526	18.136	19.738	18.852	18.997	NA
High	***P47963***	0.2405	1	6	4	1	18.885	18.935	16.013	17.293	16.409	15.300	NA
High	***P50247***	0.8838	11	88	30	11	20.923	21.160	22.477	21.052	20.787	22.396	NA
High	***P50543***	0.2767	1	6	16	1	17.752	17.485	14.160	14.952	15.663	13.599	NA
High	***P50608***	0.1349	4	42	9	4	21.699	21.271	20.135	19.574	19.824	20.487	NA
High	***P51885***	0.1551	5	65	17	5	23.357	23.554	22.978	23.838	23.960	23.378	NA
High	***P56480***	0.7720	10	35	24	10	21.499	17.648	22.748	20.686	20.078	22.782	NA
High	***P60867***	0.4669	1	6	9	1	17.755	16.605	17.550	15.461	16.727	17.813	NA
High	***P62082***	0.1437	2	7	17	2	20.769	20.769	17.069	16.015	16.339	18.053	NA
High	***P62827***	0.2608	6	39	33	6	21.879	21.473	23.813	23.189	23.537	23.771	NA
High	***P62962***	0.4644	6	52	53	6	23.127	22.773	23.876	23.852	24.000	23.010	NA
High	***P63276***	0.9505	1	4	7	1	19.748	19.445	20.267	21.094	19.864	18.656	NA
High	***P67984***	0.0785	1	2	9	1	19.335	19.121	17.474	17.111	17.302	16.301	NA
High	***P68033***	0.1924	17	337	41	2	20.702	18.526	22.245	22.221	22.766	22.696	NA
High	***P68372***	0.5944	16	175	50	2	23.866	22.742	20.433	21.038	20.963	22.932	NA
High	***P82198***	0.8014	8	76	12	8	22.061	22.072	22.238	22.995	22.228	21.517	NA
High	***P97298***	0.9423	5	90	14	5	24.314	24.443	23.293	24.679	23.828	23.654	NA
High	***P97326***	0.4077	2	16	3	2	17.930	18.063	18.262	18.135	17.924	17.826	NA
High	***Q00623***	0.4038	6	88	11	6	26.028	25.965	26.687	26.826	26.767	26.044	NA
High	***Q01149***	0.1008	3	38	3	3	21.715	22.209	21.476	21.476	21.172	21.042	NA
High	***Q01339***	0.1578	1	16	3	1	22.634	22.369	23.450	23.083	24.579	23.669	NA
High	***Q01853***	0.7968	9	35	15	9	16.426	19.437	18.444	17.775	15.914	19.492	NA
High	***Q02053***	0.2450	6	27	7	6	25.933	25.071	17.239	17.783	18.189	18.798	NA

Supporting Materials

High	*Q03265*	0.0564	17	134	51	17	22.753	21.309	20.735	22.899	25.185	25.905	NA
High	*Q04857*	0.0736	5	66	5	5	22.149	22.141	21.808	22.710	22.826	22.228	NA
High	*Q06890*	0.6767	24	322	38	24	25.463	25.184	27.080	26.347	25.600	26.709	NA
High	*Q07113*	0.2946	1	4	0	1	20.723	21.857	23.521	22.745	23.515	23.253	NA
High	*Q08761*	0.4102	1	4	1	1	19.810	17.149	20.013	20.262	19.941	19.633	NA
High	*Q08879*	0.7664	4	73	4	4	25.650	25.853	25.468	25.853	26.010	24.669	NA
High	*Q11136*	0.9855	2	6	4	2	17.442	16.803	17.107	19.075	15.775	16.439	NA
High	*Q3TPE1*	0.0960	2	20	8	2	20.589	21.234	19.777	19.948	19.293	18.519	NA
High	*Q3TTY5*	0.2782	10	125	8	7	21.953	22.530	24.047	25.780	24.872	22.438	NA
High	*Q3UV17*	0.2793	3	22	4	1	21.924	22.329	23.413	26.037	25.051	21.959	NA
High	*Q3V117*	0.5547	2	10	2	2	16.931	16.776	14.364	16.930	15.937	17.020	NA
High	*Q4FZE8*	0.0312	7	70	47	1	21.377	21.608	20.217	22.215	23.645	23.263	NA
High	*Q5FW60*	0.0378	8	66	47	4	21.507	21.917	19.906	22.579	24.590	24.015	NA
High	*Q61414*	0.1834	5	68	8	1	27.081	27.081	23.828	24.295	24.877	21.079	NA
High	*Q61599*	0.3423	1	4	4	1	16.093	17.209	17.089	18.320	18.030	16.378	NA
High	*Q61646*	0.6687	7	34	22	7	20.822	21.006	22.286	20.577	20.642	21.993	NA
High	*Q61703*	0.7741	20	513	19	20	28.547	28.659	28.600	29.012	28.918	28.147	NA
High	*Q61838*	0.9166	2	36	1	2	23.783	24.158	24.025	24.096	24.631	23.074	NA
High	*Q61937*	0.6280	3	13	10	3	18.355	18.518	18.502	16.927	19.650	17.359	NA
High	*Q62009*	0.5621	11	140	13	11	24.657	23.034	23.010	23.289	23.788	22.291	NA
High	*Q64433*	0.4865	5	24	50	5	20.113	19.790	20.817	18.318	19.530	20.915	NA
High	*Q64727*	0.2378	36	217	44	36	23.889	23.924	23.873	24.360	24.019	23.944	NA
High	*Q68FD5*	0.9965	6	17	5	6	17.627	17.329	15.930	15.757	17.966	17.151	NA
High	*Q6GQT1*	0.2759	3	234	2	3	27.284	27.344	27.423	28.416	27.491	27.504	NA
High	*Q6IFX2*	0.8254	4	22	8	2	17.567	18.859	18.560	19.604	19.302	14.921	NA
High	*Q76MZ3*	0.4227	7	24	16	7	17.274	19.027	19.712	17.005	16.786	19.312	NA
High	*Q8BMS1*	0.2882	6	18	12	6	19.094	20.377	20.203	15.975	18.917	19.820	NA
High	*Q8BND5*	0.2696	2	25	3	2	18.585	17.639	19.882	19.700	19.628	19.726	NA
High	*Q8BWT1*	0.5489	11	94	42	11	19.728	19.447	22.901	19.795	22.242	22.901	NA
High	*Q8C196*	0.7413	48	350	45	48	23.691	23.853	25.786	23.777	24.769	25.715	NA
High	*Q8K0E8*	0.3618	2	28	4	2	20.043	18.449	20.538	20.555	21.600	19.547	NA

Supporting Materials

High	***Q8R121***	0.5146	2	26	2	2	25.059	25.237	24.996	25.100	25.226	24.204	NA
High	***Q8R4U7***	0.2177	1	6	1	1	24.521	24.912	26.248	25.881	26.585	25.943	NA
High	***Q8VCM7***	0.3328	2	16	4	2	19.978	19.961	19.855	20.167	19.269	18.226	NA
High	***Q8VCW8***	0.2256	2	4	4	2	18.540	16.715	16.866	21.949	17.496	19.282	NA
High	***Q8VED5***	0.1390	6	70	7	1	20.093	20.353	22.023	23.177	24.183	21.193	NA
High	***Q91VB8***	0.5475	5	90	29	5	27.797	28.010	27.358	27.754	27.797	26.939	NA
High	***Q91VW3***	0.0651	1	15	11	1	17.850	18.065	18.103	18.865	18.656	18.254	NA
High	***Q91X72***	0.7744	5	24	12	5	16.941	17.121	20.975	18.250	17.383	20.975	NA
High	***Q91Y97***	0.5442	11	84	30	10	22.207	22.148	23.460	22.768	22.779	23.219	NA
High	***Q922R8***	0.9507	3	14	9	3	18.337	17.754	18.909	17.774	19.953	17.446	NA
High	***Q922U2***	0.2357	12	125	15	4	22.310	22.757	23.864	25.433	25.024	22.717	NA
High	***Q99JI6***	0.2609	3	19	18	3	19.274	17.696	19.878	19.860	20.179	19.821	NA
High	***Q99JY9***	0.4581	1	4	2	1	25.741	25.845	26.758	26.787	26.816	25.853	NA
High	***Q99LB7***	0.5345	5	20	7	5	16.406	19.087	20.544	18.643	20.143	20.040	NA
High	***Q99LC5***	0.2648	6	26	23	6	20.205	20.429	21.236	17.991	16.676	21.147	NA
High	***Q99PT1***	0.9626	3	26	18	3	25.065	25.102	25.028	25.264	25.591	24.278	NA
High	***Q9CQI6***	0.5884	2	16	12	2	20.457	19.218	21.261	20.890	21.134	20.123	NA
High	***Q9CZU6***	0.8390	2	6	5	2	19.320	17.929	19.630	19.538	18.777	18.947	NA
High	***Q9D8N0***	0.8978	7	32	17	7	22.791	22.771	22.213	22.753	23.103	22.067	NA
High	***Q9DBJ1***	0.5632	9	101	45	9	23.167	23.084	21.019	21.909	21.616	22.277	NA
High	***Q9ET01***	0.5836	4	12	4	4	17.810	18.868	20.142	19.859	20.385	18.243	NA
High	***Q9JJN5***	0.2229	3	67	8	3	21.097	21.341	22.274	22.345	22.098	22.125	NA
High	***Q9QUK9***	0.3682	2	168	10	2	28.511	28.803	25.609	27.373	31.080	28.972	NA
High	***Q9R098***	0.5011	3	48	5	3	21.877	21.949	21.317	22.223	22.334	21.391	NA
High	***Q9R0G6***	0.1611	11	140	17	8	24.927	25.239	24.249	24.525	24.114	23.175	NA
High	***Q9R0H0***	0.8455	8	34	18	8	21.584	18.194	21.858	17.999	21.146	21.483	NA
High	***Q9WTR5***	0.7168	5	35	10	5	20.527	20.794	19.351	20.247	19.673	20.172	NA
High	***Q9WUU7***	0.8692	2	16	4	2	19.338	19.478	20.069	20.787	19.303	18.395	NA
High	***Q9WVA4***	0.8893	4	36	29	4	20.277	20.794	20.662	20.482	20.365	20.996	NA
High	***Q9Z0K8***	0.6656	1	38	3	1	23.616	23.857	22.345	24.430	23.551	22.774	NA
High	***Q9Z1R3***	0.6107	1	36	4	1	20.516	21.137	21.165	21.679	20.707	19.015	NA

Supporting Materials

High	***Q9Z1R9***	0.4356	2	653	10	2	31.807	32.057	30.008	30.994	31.239	29.378	NA
High	***Q9Z1T2***	0.6961	8	132	9	4	22.564	22.807	22.557	23.641	22.998	21.958	NA
High	***S4R1N6***	0.7895	2	4	18	2	16.693	16.834	16.054	18.290	15.577	16.461	NA
Medium	***A0A0N4SUN5***	0.6573	1	15	4	1	21.479	21.774	17.657	19.326	20.723	18.703	NA
Medium	***A0A0R4J175***	0.4035	1	20	1	1	23.003	23.039	23.339	23.903	23.972	22.755	NA
Medium	***D3Z4W5***	0.3915	1	4	4	1	17.957	19.584	19.846	21.200	20.027	18.794	NA
Medium	***D6RI51***	0.9200	1	9	5	1	25.324	25.598	25.482	25.776	25.989	24.477	NA
Medium	***E0CYH7***	0.6154	1	2	1	1	21.584	22.017	17.832	20.436	20.700	17.562	NA
Medium	***E9PXF3***	0.5649	1	2	4	1	17.439	18.339	19.241	19.977	18.013	18.509	NA
Medium	***E9Q5F1***	0.3000	1	2	8	1	21.250	21.250	16.986	18.797	18.364	16.292	NA
Medium	***P28843***	0.3655	1	2	1	1	20.265	20.450	21.217	21.727	20.586	20.979	NA
Medium	***P35979***	0.1785	1	4	5	1	18.249	16.922	17.030	16.993	16.239	16.453	NA
Medium	***P47757***	0.4433	1	2	3	1	18.157	18.058	19.733	17.441	13.861	19.733	NA
Medium	***P55284***	0.1747	1	4	1	1	20.266	20.407	19.768	21.282	20.407	20.481	NA
Medium	***P62715***	0.9729	1	2	3	1	20.236	20.541	21.108	20.443	20.123	21.272	NA
Medium	***Q80XR6***	0.4115	1	2	4	1	21.878	21.800	19.454	22.207	21.652	21.734	NA
Medium	***Q9DD20***	0.4957	1	4	3	1	19.661	19.278	19.107	20.240	18.395	17.227	NA
Medium	***V9GX81***	0.1090	1	14	1	1	31.178	31.227	31.227	31.095	31.085	30.875	NA

Supporting Materials

Chemicals

S.No.	Chemical	Company	Cat. No.
1.	Insulin	Biorad	10516
2.	lipofectamine 3000	Invitrogen	L3000015
3.	lipofectamine RNAiMAX	Invitrogen	13778150
4.	BCA assay	Thermo Scientific	23227
5.	Nuclear and cytoplasmic extraction kit	Thermo	78833
6.	ORO	Sigma	O0625-25G
7.	Bouin's solution	Sigma	MFCD00146169
8.	Fluoroshield™ with DAPI	Sigma	F6057-20ML
9.	Amicon Ultra-15 centrifugal filter	Merck	UFC901008
10.	Gadolinium(III) chloride hexahydrate	Sigma	G7532
11.	AG 1024	Sigma	121767
12.	BODIPY	Thermo	D3922

Antibodies

S.No.	Antibody	Company	Cat. No.
1.	STK38	ABNOVA	H00011329M02
2.	pAMPKα	CST	2535s
3.	tAMPKα	CST	2532
4.	pACC	CST	3661
5.	tACC	CST	3676
6.	GAPDH	CST	d16h11(5174)
7.	α −Tubulin	CST	2144

8.	Lamin a/c	CST	4777s
9.	F4/80	CST	70076
10.	Lkb1	Abclonal	A22636
11.	Vimentin	Abclonal	A19607
12.	Alexa-647-anti-mouse IgG	Jackson ImmunoResearch	115-605-006
